# Light-responsive polymeric nanoparticles for retinal drug delivery: design cues, challenges and future perspectives

**DOI:** 10.1016/j.heliyon.2024.e26616

**Published:** 2024-02-18

**Authors:** Lorenzo Guidi, Maria Grazia Cascone, Elisabetta Rosellini

**Affiliations:** Department of Civil and Industrial Engineering, University of Pisa, Largo Lucio Lazzarino 1, 56122, Pisa, Italy

**Keywords:** Retinal diseases, Ocular nanomedicine, Polymeric nanoparticles, Light-triggered release

## Abstract

A multitude of sight-threatening retinal diseases, affecting hundreds of millions around the globe, lack effective pharmacological treatments due to ocular barriers and common drug delivery limitations. Polymeric nanoparticles (PNPs) are versatile drug carriers with sustained drug release profiles and tunable physicochemical properties which have been explored for ocular drug delivery to both anterior and posterior ocular tissues. PNPs can incorporate a wide range of drugs and overcome the challenges of conventional retinal drug delivery. Moreover, PNPs can be engineered to respond to specific stimuli such as ultraviolet, visible, or near-infrared light, and allow precise spatiotemporal control of the drug release, enabling tailored treatment regimens and reducing the number of required administrations.

The objective of this study is to emphasize the therapeutic potential of light-triggered drug-loaded polymeric nanoparticles to treat retinal diseases through an exploration of ocular pathologies, challenges in drug delivery, current production methodologies and recent applications.

Despite challenges, light-responsive PNPs hold the promise of substantially enhancing the treatment landscape for ocular diseases, aiming for an improved quality of life for patients.

## Highlights

• The ocular structure hinders effective drug delivery to the posterior eye segment.
• Polymeric nanoparticles provide a customizable solution to overcome ocular barriers.
• Light-responsive nanoparticles can enable precise drug control for tailored treatments.

## Graphical abstract

**Figure:**
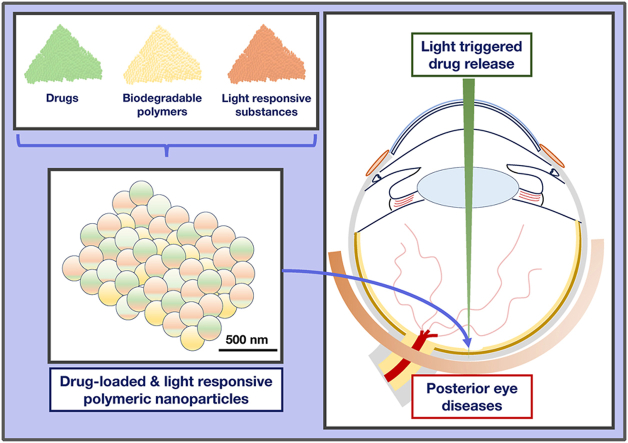

## 1 Introduction

Sight is a crucial human sense since it provides more than 75% of the information we can perceive [1]. Various degenerative ocular diseases (i.e., glaucoma, age-related macular degeneration (AMD), etc.) can lead to permanent sight loss, and currently no proven therapy can address the underlying pathologies. Moreover, tissue barriers and defence mechanisms hinder the possibility of achieving high bioavailability profiles and therefore an effective drug delivery to treat the astounding number of ocular disease cases worldwide [[2], [3], [4]]. In fact, as of the year 2020, approximately 2.2 billion individuals worldwide suffer from vision impairment, with over 200 million people moderately to severely affected [5,6]. In terms of numbers, retinal diseases pose a significant challenge, with approximately 196 million individuals affected by AMD, 146 million by diabetic retinopathy (DR), and 76 million by glaucoma. Nonetheless, in the decades to come, the demand for eye care is expected to rise significantly, substantially challenging healthcare systems [7]. Common therapies include eye drops, and subconjunctival or intravitreal injections, which result in short-term relief unless frequently administered, and possibly patient incompliance [8]. Moreover, current treatments may present many side effects or challenges in long-term safety, production, integration in the host, and resource harvesting [9]. Owing to their escalating prevalence, vision disorders are emerging as a predominant contributor to global disability. In response to this concerning trend, substantial research endeavours are being directed towards devising cost-effective, patient-suitable and sophisticated drug delivery systems (DDSs) with minimum adverse effects [5,10].

“Consider the possibility that we too can make a thing very small which does what we want - that we can manufacture an object that manoeuvres at that level!“. With these words, Dr Richard Feynman introduced the concept of nanomedicine almost seventy years ago [11]. Nanosystems exhibit a diverse range of configurations (2D and 3D), compositions, and classifications, including carbon-based materials, polymeric constructs, silica oxides, metal oxides, nanocrystals, lipids, and quantum dots [12,13]. In recent years, there has been a growing interest within the fields of pharmacology, biochemistry, and materials engineering, concerning the development and application of nanoparticles (NPs) for ocular drug delivery. To comprehensively investigate this field, three distinct searches were conducted on ScienceDirect including works between 2019 and 2023. These searches specifically targeted articles related to nanoparticles and ocular drug delivery, with a focus on three different nanoparticle types—polymeric, lipidic, and inorganic. The search queries excluded hybrid compositions. The results of these searches indicated 316 publications related to polymeric nanoparticles (PNPs), 108 focused on lipid nanoparticles, 33 on gold nanoparticles, and 31 on carbon nanoparticles.

The research on PNPs has grown to play an important role in the fields of electronics, sensors, medicine, and biotechnology. PNPs add a set of advantages to NP properties, such as biocompatibility, biodegradability, structural manipulability, enhanced solubility and stability and reduced toxicity [[14], [15], [16]]. Polymeric nanoparticles, formulated through diverse methods, can incorporate a range of chemical drug classes, proteins, and DNA, and hold great promise in addressing challenges associated with traditional ocular drug delivery systems [[15], [16], [17], [18]]. PNPs have in fact demonstrated sustained drug release and improved drug transport across ocular tissues, thereby enhancing bioavailability for the non-invasive treatment of ocular diseases [19]. Notably, PNPs have been explored as carriers for ocular drug delivery to both anterior and posterior ocular tissues, suggesting their potential to address a broad spectrum of ocular diseases [[20], [21], [22], [23]]. Moreover, the production of stimuli-responsive nanoformulations is gathering interest thanks to the possibility of triggering the release of a loaded cargo by modulating variables such as temperature, pH, ions, enzymes, and light [[24], [25], [26]]. Stimuli-responsive DDSs can present extended drug residence time, enhanced targeting, and exhibit controlled release based on stimulation intensity and duration, paving the way for patient-tailored ocular applications [[27], [28], [29]]. Light-responsive materials are especially intriguing for ocular drug release, as the eye's transparency allows the penetration of various light wavelengths, potentially overcoming limitations of endogenous stimuli like pH, temperature, and ions [25,[30], [31], [32], [33]]. Considering these features, polymeric nanoparticles emerge as a promising solution for overcoming the inherent challenges of conventional retinal drug delivery, thereby holding the potential to significantly enhance the treatment of various ocular diseases [[34], [35], [36], [37], [38]].

Considering the significance of the targeted and controlled release of therapeutic agents within the ocular domain to treat retinal diseases and its potential to garner heightened attention in future research, this review aims to provide an updated overview of the evolving landscape of light-triggered drug release from polymeric nanoparticles. The rationale of this investigation is to discuss ocular pathologies and conventional ocular drug delivery issues, illustrating ocular barriers, defence mechanisms, and common drug delivery routes adopted, highlighting their obstacles, and advancing the understanding of contemporary methodologies employed in nanoparticle production. This study will also explore the outcomes of recent applications, while possibly catalysing prospective investigations in the realm of ocular drug delivery systems. To the best of our knowledge, no other recent reviews focus on the light-responsive release of drugs from PNPs for ocular applications.

## 2 Article search methodology

⁃ **Search Approach**
  o The electronic databases employed in this study were Science Direct, Scopus, and Google Scholar.
  o The search terms/Boolean operators were: (“light-responsive”) AND (“polymeric nanoparticles”) AND (“eye”).
  o The search was limited to articles published from 2015 onwards to ensure contemporary relevance.
⁃ **Inclusion Criteria**
  o Articles concentrating on the development and applications of light-responsive polymeric nanoparticles for ocular drug delivery.
  o Studies addressing in vitro and/or *in vivo* applications.
  o Full-text articles accessible in the English language.
⁃ **Exclusion Criteria**
  o Articles where the light-responsiveness was not an inherent property of the nanoparticles, either within the polymer structure or through additional moieties, were excluded.
⁃ **Screening Process**
  o Initial screening involved a review of titles and abstracts for relevance.
⁃ **Assessment of Full Texts**
  o The complete texts of selected articles were evaluated based on synthesis methods, characterization techniques, and applications of light-responsive polymeric nanoparticles in ocular drug delivery.
⁃ **Data Extraction**
  o Extracted data encompassing nanoparticle types, synthesis approaches, responsive mechanisms, drug loading, in vitro/*in vivo* models, and demonstrated efficacy in ocular drug delivery.
⁃ **Quality Assessment**
  o Mitigated potential biases by exclusively considering peer-reviewed articles from reputable journals.
⁃ **Data Synthesis**
  o Thematic approach for information synthesis, categorizing articles according to key aspects such as nanoparticle types, responsive mechanisms, and envisioned applications in ocular drug delivery.
⁃ **Timeline**
  o The review writing process spanned over three months, starting from the initial database searches to the conclusive data synthesis.

## 3 Conventional ocular drug delivery

Moderate to severe visual impairment affects more than 250 million people to this date and the number of blind individuals is projected to reach 115 million by 2050 if treatments are not improved [39]. Of the several treatment options available for managing ocular diseases, medication administration is the most common method. However, ophthalmic drug delivery involves some of the most difficult routes of drug administration, in both human and veterinary medicine. Its drawbacks include short ocular retention time, reduced drug accumulation, and insufficient bioavailability, leading to limited therapeutic benefits. Although easily accessible, the eyes are protected by various barriers and defence mechanisms [[39], [40], [41], [42]]. The following sections will give an insight into ocular anatomy and pathology, drug administration routes, and the barriers that hinder a successful therapy.

### 3.1 Ocular anatomy and retinal diseases

**Fig. 1:**
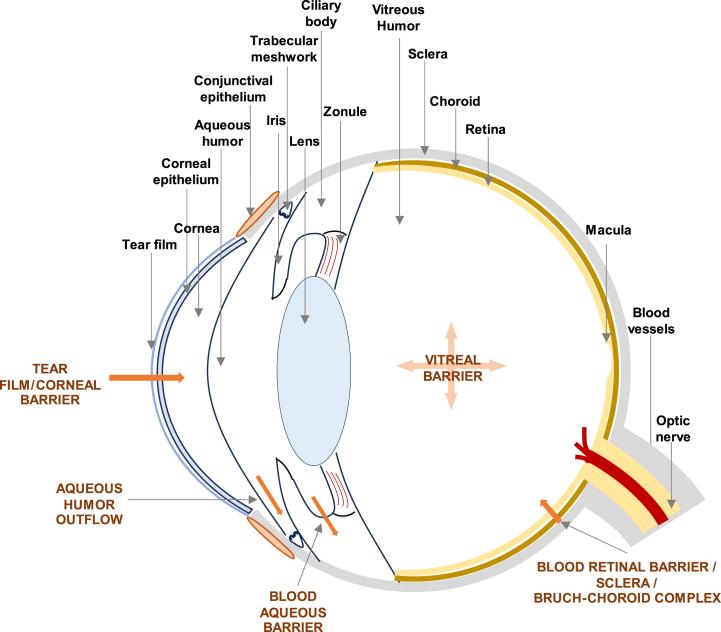
A schematic depiction of ocular anatomy and its main delivery barriers, as detailed in the previous paragraphs.

As depicted in Fig. 1, the eyes are anatomically divided into the anterior and posterior segments. The anterior segment consists of the cornea, aqueous humour (AH), conjunctiva, iris, and lens, while the posterior segment comprises the retina-choroid, sclera, optic nerve, and vitreous humour [39]. The anterior and posterior fluid chambers are filled with aqueous humour that nourishes the cornea and lens, while the vitreous chamber contains vitreous humour. The retina consists of three nuclear layers and two synaptic layers, with the photoreceptors located in the outer nuclear layer. In contrast, the inner layers contain the axons of the photoreceptors, the dendrites of the bipolar and horizontal cells (outer plexiform layer), the nuclei of the bipolar, horizontal, and amacrine cells (inner nuclear layer), the ganglion cells' axons synapse with the bipolar and amacrine cells' dendrites (inner plexiform layer), the ganglion cell layer and the nerve fibre layer (the innermost layer of the retina). The ganglion cells' axons form the optic nerve, which eventually sends information to the visual cortex [1,43].

Several diseases targeting ocular districts are currently being addressed by pharmacological treatments. Among these, retinal degenerative diseases are one of the most common causes of visual impairment and may lead to blindness. Drugs currently available cannot restore lost cells or prevent damaged cells from dying, being able only to control the conditions that endanger the tissues (i.e., intraocular pressure control eye drops to prevent retinal ganglion cell death from Glaucoma) [6,44,45].

Glaucoma is one of the most frequent irreversible blindness-causing diseases, where intraocular pressure has been identified as the only modifiable risk factor. It is associated with an imbalance in aqueous humour production and reduced drainage due to abnormal trabecular meshwork and Schlemm's canal, possibly caused by connective tissue growth factor (primary glaucoma). Glaucoma leads to a gradual vision loss due to damage to the retinal ganglion cell layer [46]. Endophthalmitis is an infectious disease caused by bacteria or fungi, resulting in permanent vision impairment in the affected eye [47]. Uveitis, characterized by inflammation of the uvea, retina, and papilla, is a major cause of visual morbidity in the working-age group, leading to moderate to severe visual impairment [48]. AMD, the third leading cause of blindness and the primary cause of irreversible vision loss, is influenced by age, genetic factors, lifestyle, cardiovascular health, and sunlight exposure. It leads to choroidal/retinal neovascularization and moderate to severe vision impairment [49]. DR, a prevalent microvascular complication of diabetes, is the primary cause of avoidable vision loss in working-age individuals, affecting approximately one-third of people with diabetes. DR is associated with hyperglycaemia, hypertension, dyslipidemia, diabetes duration, and cataract surgery, leading to retinal nonperfusion, ischemia, and vision loss [50]. Retinal neovascularization, characterized by the unregulated growth of existing blood vessels, is associated with AMD and DR, resulting in moderate to severe vision impairment [51]. Choroidal neovascularization (CNV) is a nonspecific wound repair response triggered by choroidal ischemia/hypoxia, leading to the abnormal growth of choroidal vessels in the sub-retinal space through Bruch's membrane [49]. Inherited retinal diseases, such as retinitis pigmentosa, encompass a group of disorders characterized by clinical and genetic diversity, leading to various forms of blindness due to genetic abnormalities [52]. Retinoblastoma, a highly aggressive eye tumour primarily affecting infants and children, is caused by sporadic or hereditary mutations of the RB1 gene. It results in loss of vision, secondary monocular tumours, and even death [53]. Uveal melanoma is the most common primary intraocular cancer that can spread, causing the patient's death. It is associated with genetic factors, UV exposure, and ocular melanocytosis [54].

There are no current effective treatments for these retinal diseases: their positioning is challenging for common eye-drop delivery due to the acellular nature of the vitreous body and the long diffusion distance, decreasing the bioavailability of a drug [55].

### 3.2 Delivery barriers

The following subparagraphs will illustrate the various barriers through which the eye impedes drug delivery, starting from the outermost tear film and progressing towards the inner barriers.

#### 3.2.1 The tear film barrier

In the anterior ocular segment, the first barrier for topical drug administration is the tear film, continuously produced and drained by the lacrimal system. The tear film is composed of three layers:

⋄ the mucous layer (primarily water with electrolytes, and surfactant mucins)
⋄ the aqueous layer (primarily water and electrolytes, along with proteins with lubrication, nourishment, and waste depletion functions)
⋄ the outermost lipid layer (reduces evaporation and prevents the drying of the eye)

The tear film acts as both a structural and dynamic barrier given the fast and periodic downflow of tears, produced at 0.5–32.2 μL/min, via the nasolacrimal duct path and their evaporation. Common eye-drop drugs are administered in ∼35 μL volumes and can reach a precorneal half-life on the eye surface between 1 and 3 min. Tear turnover results in up to 85% of the drug applied onto the ocular surface being eliminated to the systemic circulation. This percentage varies with the patient's blinking rate, the physicochemical properties of the drug, and its lipophilicity. Other factors affecting the bioavailability of a drug are the binding and metabolism by aqueous layer proteins (globulin, albumin, and lactoferrin) or drug trapping in the mucus layer, which contains negatively charged glycans and hydrophobic regions in its porous structure.

The molecules that manage to cross the tear film barrier can travel through either the corneal pathway, which involves crossing the cornea, aqueous humour, lens, vitreous humour, and retina, or the non-corneal pathway, which involves crossing the conjunctiva, sclera, choroid, and retina [1,39,42,56].

##### 3.2.1.1 The corneal pathway

In the anterior segment, the rapid turnover (∼2–3 μL/min) of the poorly viscous AH (∼300 μl), produced by the ciliary epithelium, represents another dynamic barrier to the ocular absorption of drugs, of which an estimated 3% reaches this level when applied topically [56]. The AH encounters the lens and the pupil along its pathway, which ends through the trabecular meshwork in the Schlemm's canal, where it is drained to the episcleral veins. The AH turnover reduces the drug concentration gradient and travels opposite the direction of the required drug molecule movement, further impacting topically applied drug efficacy [42,56].

Travelling towards the posterior segment from the AH, drugs come across the iris, the pupil, and the lens. Scarce data do not allow to determine the barrier nature of these structures [39,57]. Nonetheless, there is proof that relevant drug permeation-hindering factors are expressed by the ciliary body and iris. Moreover, the melanin pigment in the ciliary body can bind to drugs, and the iris and lens structures may obstruct drug penetration to the posterior segment [42,56,58,59].

##### 3.2.1.2 The non-corneal pathway

This route comprises passages through the conjunctiva, the sclera, and the choroid. The conjunctiva is a thin transparent mucous membrane lining the inner surface of the eyelids and covering the sclera up to the corneal margins. It consists of an upper epithelium and a lower stromal layer (substantia propria), which contains numerous blood and lymphatic vessels [42,56,58,59].

The conjunctival epithelium's permeability is 25 times higher than that of the corneal epithelium due to its wider surface area (17 times), fewer layers of epithelial cells, and larger paracellular spaces (250 times), making it more permeable, especially for large hydrophilic molecules. However, drugs that penetrate the conjunctiva may enter the general circulation through the conjunctival sac or nasal cavity, leading to significant drug loss, particularly for smaller hydrophilic molecules, which can decrease their ocular bioavailability [42,57].

The conjunctival stroma is an important barrier to drug penetration after topical or periocular administration. In particular, the size of the drug molecule affects the extent of its clearance. Small hydrophilic molecules are more likely to be taken up by conjunctival blood vessels and cleared to the systemic circulation by passing through capillary fenestrations, which range in size from 30 to 100 nm, resulting in a reduction in drug bioavailability [56]. Compared to the cornea, the conjunctiva is more conducive to the absorption of large and hydrophilic molecules such as small interfering RNA and peptides [39,60].

To sum up, the tear film and the cornea, together with the blood-aqueous barrier (BAB), which will be discussed in paragraph 3.2.3.4, represent the primary hindrances in the front part of the eye. The AH and lens can also impede drug delivery to the posterior eye [39].

#### 3.2.2 The cornea and anterior segments

The cornea is the most prominent physical and chemical barrier to paracellular drug permeability. Molecular size, weight, lipophilicity, and charge affect drug permeability through this barrier [56]. This segment is a transparent multi-layered membrane consisting of both cellular and acellular layers:

⋄ the corneal endothelium (CEn), the innermost layer, is made of basal, wing, and superficial cells held together by strong intracellular junctions. These tight junctions can determine a cut-off molecule size down to 5.5 Å. The CEn accounts for up to 90% of the barrier to hydrophilic drugs and 10% to lipophilic drugs. Moreover, drug efflux pumps and drug-degrading enzymes hinder drug transport across this layer [1,39,42,56].
⋄ the stroma, predominantly composed of water, crosslinked collagen fibrils, and interfibrillar carbohydrate molecules and populated by keratocytes, inhibits the permeation of highly lipophilic molecules but allows the permeation of hydrophilic drugs [1,39,42,56].
⋄ the corneal epithelium (CEp), the outermost layer, is important for water regulation in the cornea, and although its permeability relies mostly on molecular size, it may offer further resistance towards lipophilic drugs. Cationic and small (log D = 2−3) lipophilic drugs can permeate the cornea more effectively. Topically applied drugs display a low rate of reaching the anterior chamber (estimated maximum at 5% for lipophilic molecules and 0.5% for hydrophilic molecules) [1,39,42,56].
⋄ the membranes (Bowman's and Descemet's) are acellular layers and do not affect drug permeation significantly [1,42].

#### 3.2.3 The posterior eye

In the posterior eye, the sclera, the choroid, the vitreous body, and the blood-retinal barrier are the main obstacles to efficient drug delivery [39].

##### 3.2.3.1 The sclera

The sclera poses a significant challenge to ocular drug delivery due to its restricted permeability. The sclera is a dense, hydrophilic connective tissue consisting of an intersecting and stacked scleral collagen matrix and a negatively charged proteoglycan matrix in the inter-fibril space. The sclera is composed of three layers, altogether covering over 80% of the surface of the eyeballs [39]:

⋄ the episclera, which is the outermost vascular layer
⋄ the stroma, a fibrous matrix responsible for most of the thickness of the sclera
⋄ the lamina fusca, which is the innermost connective tissue layer

The thickness of the sclera and the fact that it covers most of the eyeball's outer shell are crucial aspects of transscleral drug delivery [39,42,56]. The ability of a drug to permeate the sclera mostly depends on its molecular weight and charge: negatively charged drugs with lower molecular weights can more effectively penetrate this barrier. Positively charged molecules have their permeation hindered by being attracted to negatively charged proteoglycans. Additionally, the episcleral lymphatic flow acts as a dynamic barrier to drug permeation [56,61].

##### 3.2.3.2 The Bruch-choroid complex

The choroid, located between the retina and the sclera, is a thin, highly vascularized and innervated dynamic barrier that supplies blood to the retina. It consists of five layers, arranged from the outermost to the innermost: the suprachoroidal cavity, two vascular layers, the choriocapillaris layer, and Bruch's membrane [42]. Its dynamic structure is responsible for a significant drug clearance to the systemic circulation, even though less significant than that via the conjunctival route [56]. Peptides and macromolecules are cleared from the body at a slower rate because they cannot easily pass through the small openings in the choriocapillaris endothelium, while the delivery of small molecules is more challenging [56].

**Fig. 2:**
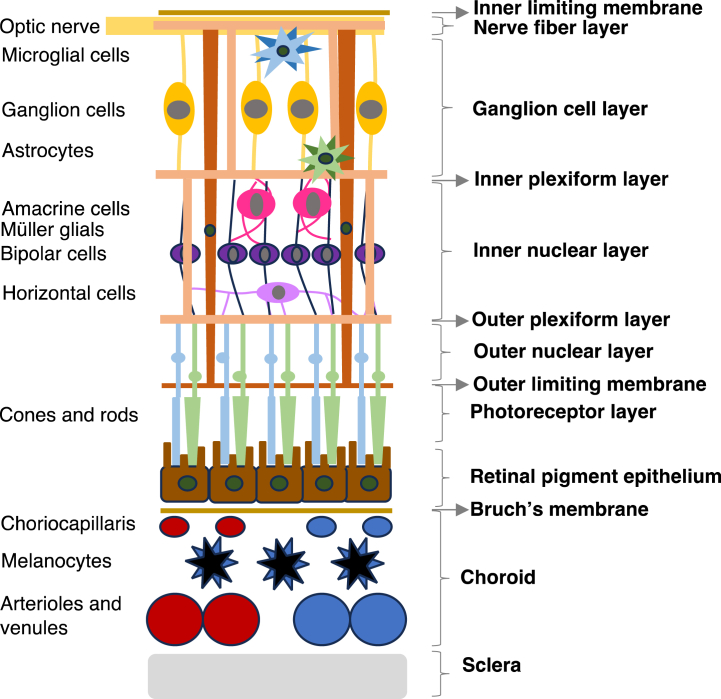
A detailed scheme of the layers constituting the retina and its neighbouring structures (the nerve fibre layer and the sclera-choroid complex).

Bruch's membrane is a thin collagenous membrane (2–4 μm), adjacent to the choriocapillaris, layer serving as the basement membrane of the retinal pigment epithelium (RPE) [42]. The Bruch-choroid complex is a more significant barrier to drug delivery through the transscleral (non-corneal) pathway than the sclera itself, as it can bind solutes, especially positively charged lipophilic drugs, which may form a slow-release drug depot in the Bruch's-choroid complex. Bruch's membrane appears to be permeable to large molecules (up to 150kD), even though permeability declines with age [56], with varying permeability with respect to molecular size [42,43,62] (Fig. 2).

##### 3.2.3.3 The vitreous body

The vitreous body, located between the lens and retina, is a jelly-like substance made up of water (99%), collagen fibrils, hyaluronic acid (HA), sulphates, and other proteins. The volume of the vitreous gel in adults is usually around 4 mL and varies with age and existing medical conditions [43,56]. Drug movement across the vitreous occurs mainly by diffusion, with minimal flow, although recent research suggests that the AH may flow through the vitreous, particularly around the retinal pigment epithelium, and exit through the choriocapillaris [56].

Vitreous humour acts as the first barrier against drug permeation to retinal and choroidal tissues for intravitreal administration routes, redirecting drugs either to the AH flow (anteriorly) or to the retinal vascular system (posteriorly). While molecules of all sizes may follow the first depletion route, clearing through the retinal pathway requires molecules to be able to diffuse through either the endothelium of the retinal blood vessels (inner blood-retinal barrier (BRB)) or the retinal pigment epithelium (outer BRB) and reach the choriocapillaris. This results in large hydrophilic molecules following anterior depletion routes, while smaller lipophilic molecules are subject to an enhanced posterior clearance, which can be considered a more effective clearing route [42,56,63]. Moreover, the viscosity of the vitreous fluid impedes the diffusion of larger and heavier therapeutic cargoes such as proteins and its net anionic charge regulates the diffusion of drug molecules (entrapping only positively charged particles) [1,64]. Therefore, molecular weight and charge of administered drugs significantly impact retinal bioavailability.

The inner limiting membrane (ILM), formed by the footplates of Müller glial cells, is a further structural barrier for retinal drug delivery (Fig. 2). It acts as a strict physical barrier against most nanosized molecules due to its average pore size of 10–25 nm, even though uptake and transcellular permeation via Müller cells may be an alternative mechanism to deliver drugs into the inner retina from the vitreous chamber [39,42,65]. Despite this, larger nanosized molecules seem to be able to penetrate the ILM and spread throughout the retina, indicating that various active penetration mechanisms may be present [42,43,56,64].

##### 3.2.3.4 The blood-eye barrier

The blood-eye barrier (BEB) is composed of the anterior blood-aqueous barrier (BAB) and the posterior blood-retinal barrier (BRB).

The BAB is comprised of tightly connected cells (iris epithelial cells, capillary endothelial cells, and non-pigmented epithelial cells of the ciliary body), that form a barrier to prevent drug entry from the plasma into the aqueous humour, hindering systemic administration. However, the fenestrated capillaries in the ciliary body stroma enable the entry of small molecules (i.e., plasma protein leakage) into the iridial circulation [39,42,56].

The BRB prevents drug diffusion into the retina from systemic circulation and it is further divided into inner and outer BRB (Fig. 2). The inner blood-retinal barrier (BRB) is made up of retinal endothelial cells, which line the microvasculature that provides blood to the neural retina. The tight intercellular junctions of the inner BRB selectively defend the retina against foreign substances in the blood (hydrophilic compounds and macromolecules) [1,42,56]. The outer BRB consists of tight junctions between RPE cells and allows only a small percentage (around 1–2%) of an administered drug to reach the retina and vitreous region [42,66]. This layer controls the movement of solutes and nutrients through the paracellular pathway from the choroid to the sub-retinal space, as well as the elimination of metabolic waste and water from the retina to the choroid [1]. As a result, small lipophilic molecules can permeate the RPE more easily than hydrophilic molecules due to their ability to diffuse through the intracellular route. Furthermore, the RPE permeability is inversely proportional to the molecular weight of a drug, with smaller molecules exhibiting higher diffusion rates. However, the barrier function of the RPE can be significantly compromised under pathological conditions. The RPE also acts as a metabolic barrier to drug permeability, as it expresses a variety of enzymes and efflux proteins [56,67,68].

### 3.3 Delivery routes

**Fig. 3:**
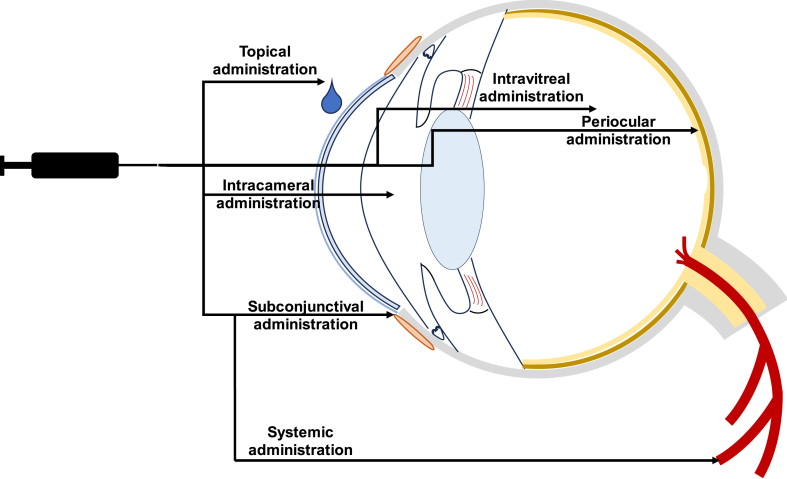
A schematic representation of the main drug delivery routes discussed in paragraph 3.3.

The crucial factor for managing retinal diseases is delivering an adequate drug dosage to the affected area. Ocular drug delivery paths are divided into topical, systemic, and injection routes (Fig. 3). These methods aim at bypassing ocular barriers and achieving the desired drug concentration in the targeted location [39,42]. Optimal drug penetration and deposition are impeded by the anatomical and physiological characteristics of both the front and back parts of the eye in all these methods [56]. The upcoming paragraphs will demonstrate the different administration routes that can be adopted for posterior-eye drug delivery.

#### 3.3.1 Topical route

The most common method of managing eye disorders is through topical application, thanks to its non-invasiveness (>90% of ophthalmic products in the market) [42]. Topical administration refers to the application of medication to the surface of the eye, including drug delivery systems (DDSs) like drug-eluting contact lenses [39]. However, topical instillations suffer from many limitations. The drug delivery efficiency is mainly limited by the loss of medication before it reaches the cornea, caused by the high rate of tear turnover, blinking, lacrimation, and nasolacrimal drainage [42,69]. As a result, eye drops must be frequently applied to maintain the required drug concentration, which can lead to poor patient compliance and complications (damage from preservatives, ocular irritation, complications from steroid use, high drug concentrations in the bloodstream, and off-target side effects). In addition, topical applications are not very effective in treating posterior eye disorders, because of the corneal barrier and consequently, they are commonly used to deliver drugs to the front part of the eye. It has been shown that only around 5% of the applied drug can penetrate the ocular surface, and even less reaches the vitreous and underlying retinal tissues [38,56,70].

#### 3.3.2 Systemic route

Systemic drug administration to ocular tissues comprises intravenous and oral dosing. This type of delivery poses significant challenges: orally administered drugs must survive the harsh environment of the gastrointestinal tract and undergo first-pass metabolism while intravenously administered drugs are hindered by the lower blood supply to the eye compared to the rest of the body, resulting in limited drug accumulation. Moreover, the BAB prevents drugs in the systemic circulation from reaching the anterior ocular tissues, while both the inner and outer BRB limit drug administration in the posterior part of the eye to a great extent. Consequently, 1–2% of the dosage reaches the target site, so achieving the desired therapeutic efficacy requires high dosages and frequent drug administration, which may result in systemic adverse effects and poor compliance with the therapy [1,39,42,56,71].

#### 3.3.3 Intraocular injections

Intraocular injections can follow intracameral, subretinal, suprachoroidal, intrastromal and intravitreal routes [42].

Intracameral administrations bypass the corneal barrier, leading to a higher aqueous humour drug concentration in comparison to eye drops. This method is commonly used for treating fungal and bacterial keratitis or after cataract surgery. This technique isn't particularly effective in delivering drugs to the back part of the eye due to the drug's difficulty in opposing the AH flow [39,42].

Intravitreal injections have become the preferred method for treating vitreoretinal diseases, allowing for the delivery of drugs directly into the back of the eye through an injection or implantable devices. Up to 100% of the administrated drug reaches the target with minimal toxicity to the front part and without the side effects typical of systemic administrations [56]). This method increases the concentration of drugs in the vitreous and retina but carries a higher risk of complications, due to the frequency of delivery needed, such as optic nerve damage, endophthalmitis, retinal detachment, raised intraocular pressure, inflammation, cataract, infection, and vitreous haemorrhage. On the other hand, intravitreal implants can deliver drugs over a long period of time (1–6 months) without multiple injections or complications. However, these systems, available in biodegradable and non-biodegradable forms, usually require one or more surgical procedures [39,42,72]. Other common limitations to these systems include lack of ocular-segment targeting (with possible side effects to healthy tissues), the possible requirement to adjust the drug dose, and the predetermined drug release rate [39,42,[72], [73], [74]].

#### 3.3.4 Periocular injections

Periocular administration refers to the delivery of drugs to the tissues surrounding the eye, such as subconjunctival, sub-tenon, peribulbar, retrobulbar, or posterior juxta scleral routes. Depending on the technique used, the drug can penetrate the sclera, choroid, retinal pigment epithelium (RPE), and neural retina [56]. For diseases affecting the subretinal space, subretinal injection is used to deliver medication directly between the photoreceptors and RPE cells [42,75,76]. The suprachoroidal administration method places drugs between the choroid and sclera, reducing the chances of systemic side effects [42,75,76]. Intrastromal injection is a minimally invasive technique used to deliver medication to the corneal stroma, without causing damage to the surrounding tissue structures [42,75,76]. Periocular administrations have the advantage of allowing for larger volumes of medication to be injected, providing a longer duration of action compared to intravitreal injections (up to 10-fold the volume injected intravitreally), with less invasiveness and a lower risk of ocular pain, infection, endophthalmitis, or bleeding [42,77]. Although the risk of corneal exposure is minimal, exposure of the sclera and conjunctiva carries a high risk of ocular toxicity. Furthermore, significant drug clearance through the vascular system of the conjunctiva can lead to an increase in systemic drug absorption [56]. Periocular injections are not immune to ocular barriers as they can be troubled by the episcleral, sclera, choroid, and Bruch's membrane [39]. Subconjunctival injections are commonly used to deliver drugs to the front part of the eye, but their bioavailability is often limited as they are absorbed by the lymphatic and circulatory systems. This results in the need for frequent injections, which can cause complications like conjunctival oedema and subconjunctival haemorrhage. The other periocular paths are mainly used for anaesthesia during eye surgeries. However, periocular administration still falls short of delivering sufficient medication to the retina, due to drug loss from the periocular space, the blood-retinal barrier, and choroidal circulation [39,42,77].

## 4 Ocular nanomedicine

As millions of people around the world are affected by many sight-threatening ocular diseases, there has been an increase in research over the past decades into the causes of these pathologies and the development of highly potent and effective pharmacological treatments. However, because of the illustrated ocular barriers and the limitations of common drug delivery routes, such as eye drops or intravitreal administrations, their clinical efficacy is currently restricted [4,78,79].

To address these issues, researchers have turned to nanomedicine, which bears the hope for improved bioavailability, drug delivery, and possesses diagnostic capabilities while minimizing unwanted side effects. While the role of this research area is still being explored, nanomedicine holds significant promise in advancing ocular disease management and improving therapeutic outcomes. The use of different types of nanomaterials has greatly expanded the range of applications in nanomedicine, especially in the field of ophthalmology. Polymeric nanoparticles made of polyester or natural biopolymers are among the most sought-after formulations [1,4,[80], [81], [82], [83], [84], [85], [86], [87]]. This section describes the materials that can be used to obtain effective and clinically relevant nanoparticle formulations, the production methods, the nanoparticle properties that need to be controlled to tailor a DDS towards various ocular districts and includes a discussion on drug-release behaviour and nanocarrier fate.

### 4.1 Polymeric nanoparticles

PNPs have gained major attention in drug delivery thanks to their enhanced stability as carriers for biological factors and to the possibility of tailoring their properties by manipulating the structure, shape, and composition of the polymer [88,89]. Several polymers have the advantage of being biodegradable, breaking down into non-toxic elements in the body. Biodegradability eliminates the need for manual removal of the drug delivery system once it has performed its function, which is crucial for ocular implant development [90]. PNPs enhance drug preservation and prolong half-life, while also mitigating drug clearance from the body. Additionally, they minimize adverse effects, enhance therapeutic efficacy, and help overcome physiological barriers by sidestepping efflux pumps, and decrease drug dispersion through healthy tissues [91].

Furthermore, PNPs offer the possibility to trigger the release of biological factors with the onset of specific stimuli, such as temperature [92], pH [93,94], or light [34,35]. There are many methods to produce PNPs, which all lead to the precipitation of a polymer [95]. In the next paragraphs, an overview of PNP fabrication methods and polymers used for this application will be provided.

#### 4.1.1 Common degradable polymers for drug delivery

The emergence of biodegradable polymers has revolutionized medicine in the past 50 years, driven by the need for longer-lasting therapeutics and reduced toxicity of small molecule drugs and leading to significant advancements in drug delivery. Synthetic biodegradable polymers have found applications in drug delivery since the 1960s. These degradable polymers are now widely used due to their biocompatibility and ability to break down in the body into non-toxic by-products like water and carbon dioxide, facilitating an easy elimination [25]. Among biodegradable synthetic polymers, polyesters such as poly (glycolic) acid (PGA), poly (lactic) acid (PLA), and poly (lactic-*co*-glycolic) acid have received the greatest attention in research [25]. Polyester nanoparticles are recognized to be tuneable and display consistent production outcomes, making them ideal for addressing a variety of medical challenges, including ocular drug delivery [4,96].

Naturally occurring polymers are also appreciated in ocular nanomedicine for their biocompatibility, biodegradability, and low toxicity. Among these, gelatin, chitosan, alginate, and, more recently, polydopamine, have been employed in some interesting studies [4]. In the next sections, an overview of the natural and synthetic polymers that are most used to produce PNPs is provided.

##### 4.1.1.1 Natural polymers

Gelatin is a water-soluble protein commonly derived from the skin, bones, and connective tissues of pigs or fish. Gelatin is one of the most biocompatible polymers and has been validated for many applications (i.e., food, pharmaceutical, and cosmetic industries) [97,98]. Moreover, it is biodegradable, economical, and displays low immunogenicity. Gelatin's degradability stems from its terminal amino residues, which can be cleaved into individual amino acids through acid or base hydrolysis. Gelatin degradation can also occur through enzymatic digestion by proteolytic enzymes (papain, pepsin, chymotrypsin, and trypsin) [99]. Its mechanical properties can be adjusted by cross-linking with glutaraldehyde or genepin, photo-crosslinking, etc. Gelatin has functional groups that can be used for cross-linking, as well as for functionalization with biomolecules. Additionally, it moisturizes and swells in an aqueous solution and degrades rapidly above its dissociation temperature Td, the value of which depends on factors such as molecular weight, amino-acidic composition, and the number/quantity of plasticizers (i.e., moisture, citrate esters, polyols, or polyethylene glycol [100]). The characteristic dissociation temperature Td is generally lower than the human body temperature [[101], [102], [103], [104], [105], [106]]. Gelatin-based nanoparticles are a versatile and promising DDS that can be customized with fluorescent dyes, hydrophilic polymers, targeting moieties, or active drugs while maintaining their essential properties [82,[107], [108], [109]]. Although some manufacturing methods can lead to toxicity, the ionic gelation method can produce cationic gelatin nanoparticles, while two-step desolvation methods can reduce particle aggregation and produce a lower polydispersity index [97]. Chang et al. investigated the use of gelatin nanoparticles loaded with EGCG and modified with RGD-HA conjugates for treating corneal neovascularization (CNV). The modified nanoparticles inhibited HUVEC migration and tube formation and exhibited enhanced uptake, showing their potential for treating CNV through topical administration [110]. A study by Mahor et al. produced positively charged moxifloxacin-loaded gelatin nanoparticles. They managed to obtain an initial burst release followed by a sustained (12 h) drug distribution for antibacterial purposes, the effectiveness of which was verified to be higher than commercially available products *in vivo* [109].

Chitosan is a highly cationic linear polysaccharide deriving from chitin, which can be found in arthropod exoskeletons [101]. It possesses mucoadhesive benefits which derive from the many functional groups it displays (hydroxyl, carboxyl, and amino groups). These mucoadhesive properties have been utilized in eye drops to increase therapeutic bioavailability, and in extended-release gels for subconjunctival injection [4]. Moreover, they allow chitosan nanoparticles to remain in ocular tissues for longer periods and prevent drug clearance [90,111,112]. Chitosan is also amphiphilic, which allows for improved solubility of hydrophobic drugs and increased penetration through the corneal membrane compared to non-conjugated drugs. Numerous publications demonstrate the loading capacity and the in vitro and *in vivo* efficacy of chitosan [4,[113], [114], [115]]. To treat DR, Lu et al. developed chitosan nanoparticles, that were loaded with bevacizumab and administered via transscleral injections, as reported in their study [116]. Lomefloxacin hydrochloride, another commonly used antibacterial agent, was loaded into chitosan nanoparticles with good encapsulation efficiency (57–69%) for ocular applications. The release of lomefloxacin hydrochloride from the nanoparticles was extended over 8 h (approximately 94% of the released drug), while the release from lomefloxacin hydrochloride powder was almost complete within 30 min [117]. In another example, Jiang et al. took advantage of chitosan's cationic properties to deliver anti-VEGF bevacizumab intravitreally, using microparticles made up of a chitosan core, with a positive charge, and a PCL shell [118].

Alginic acid is a block copolymer of natural anionic polysaccharides found in brown algae cell walls and certain bacterial strains. Although alginic acid is insoluble in aqueous/organic solutions, alginate monovalent salts and esters, such as the highly swelling and negatively charged sodium alginate, form viscous solutions with water [119,120]. Increasing the concentration of alginic acid significantly increases its viscosity (a 0.5% w/w aqueous dispersion has a viscosity of 20 mPa*s, while a 2% w/w aqueous dispersion shows a much higher viscosity of 2000 mPa*s) while an increase of temperature decreases viscosity [120]. The polymer's backbone consists of varying ratios of β-D-mannuronate (M) and α-l-guluronate (G) residues, with the latter responsive to physical cross-linking with divalent cations [119,[121], [122], [123]]. Ionotropic gelation occurs when sodium alginate is exposed to different aqueous divalent cations, such as Zn^2+^, Ba^2+^, and Ca^2+^ [120]. Alginate naturally degrades slowly and uncontrollably, releasing high molecular weight strands that are challenging to eliminate from the body. However, limited oxidation of alginate with periodate enhances its hydrolysis in aqueous solutions. This modified alginate degrades at a rate determined by the pH and temperature of the solution while maintaining its ability to form calcium-ion cross-linked gels [124]. Costa et al. developed chitosan-alginate-coated nanoparticles for delivering daptomycin into the eye, which had encapsulation efficiency values of up to 92%. The in vitro ocular permeability of the nanoparticles was found to be 9%–12% over 4 h into a retinal cell monolayer after the drug was administered [125]. Kianersi et al. prepared alginate nanoparticles to deliver betamethasone sodium phosphate, an anti-inflammatory drug, to the eye. The encapsulation efficiency and loading capacity of the alginate nanoparticles were approximately 40% and 7%, respectively. *In vitro* drug release assays demonstrated sustained drug release over 120 h for both uncoated and coated samples. These findings suggest that the use of alginate nanoparticles may help improve the quality of life for patients suffering from eye diseases, by reducing the need for multiple doses, as compared to commercial drug drops [126].

Finally, polydopamine (PDOPA) is a biopolymer that has gained significant attention in drug delivery due to its biocompatibility and low toxicity. It is formed through the oxidative polymerization of dopamine, a major neurotransmitter in the human body. Dopamine can also be derived from l-DOPA, an intermediate amino acid found in marine mussels deemed to be responsible for their strong adhesive capabilities. Researchers have investigated dopamine as a possible biomimetic material since the beginning of the last century. PDOPA has since been used for coatings and nanostructures, with recent studies focusing on its potential in ocular drug delivery [115,[127], [128], [129], [130]]. PDOPA exhibits NIR-responsive features, which are attractive for on-demand drug delivery applications and is relatively facile and economical to produce while demonstrating small and monodisperse nanoparticle populations [129,[131], [132], [133]]. Finally, drugs can easily be adsorbed on the surface of PDOPA nanoparticles by π-π stacking or hydrogen bonding and the catechol and anthracene fractions allow secondary modification with thiol or amino group-containing compounds (i.e., polyethylene glycol (PEG)-SH or PEG-NH_2_) to improve the half-life or cellular uptake of a nanoparticle population [115,127,[134], [135], [136]]. For example, Jiang et al. developed PDOPA nanoparticles that exhibited effective loading and release of anti-VEGF in vitro in an oxidative stress-dependent manner. Recent research has also shown that PDOPA coatings can enhance nanoparticle mucopenetration and cellular uptake compared to uncoated nanoparticles [137]. Lou et al. designed a PDOPA-based nano platform to protect retinal ganglion cells from the damage stemming from oxidative stress, which leads to the death of these cells in many diseases (ocular trauma, Glaucoma, etc.). PDOPA exhibited reactive oxygen species (ROS)-scavenging effects and was able to alleviate injury and inflammation in vitro and in an optic nerve crush model with a single intravitreal injection. Moreover, brimonidine, a neuroprotective drug, was delivered through PDOPA NPs to amplify the anti-inflammatory properties and promote axon regeneration whilst attenuating retinal ganglion cell (RGC) loss [128].

##### 4.1.1.2 Polyesters

PLGA is a synthetic copolymer consisting of PGA, a hydrophilic, highly crystalline polymer that degrades quickly, and PLA, similar in structure, but with distinct chemical, physical, and mechanical properties due to a methyl group on the alpha carbon. PLGA is produced through ring-opening polymerization (ROP), which involves using cyclic lactide (L) and glycolide (G) monomers. PLGA offers a wide range of degradation rates based on its monomer ratios, crystallinity, and chain composition [138]. Once formed, PLGA can degrade when its ester bonds are hydrolysed in water [25]. A more pronounced concentration of L monomer decreases the degradation rate of PLGA [139]. To enhance the biocompatibility and *in vivo* circulation of PLGA NPs, researchers developed block copolymers of PLGA and PEG (PLGA-PEG). Hydrophilic PEG can position itself towards the surface of polymeric NPs, leading to increased hydration [140]. PLGA nanoparticles are particularly popular because they are non-toxic, biodegradable, and can effectively load and selectively and sustainably release various types of drugs, from small hydrophobic/hydrophilic molecules to large biopharmaceuticals, making them an ideal option for efficient drug delivery and long-term release in ophthalmology [141]. Drug release from polyesters like PLGA or PLA is not solely dependent on degradation (more prominent in the later stage of release), but also on diffusion of the drug under concentration gradients [26]. For instance, Jiang et al. developed PLGA nanoparticles with chitosan and trehalose surface modifications, achieving optimal drug release and permeability with a 50:50 lactide:glycolide ratio and low molecular weight (10 kDa), showing high entrapment efficiency, *trans*-ocular permeation, and retention [142]. The precisely controlled chemical structures and biological inertness of synthetic polymers, which make them highly versatile and uniform, come at the cost of biological cues, which lack giving their biologically inert characteristics. Therefore, it is important to incorporate functionalities into the polyester structure to enhance interactions with targeted cells and tissues [4,141]. In another example, researchers fabricated chitosan-coated PLGA nanoparticles loaded with bevacizumab for the treatment of posterior ocular diseases. The chitosan coating on the PLGA nanoparticles resulted in improved mucoadhesion to the sclera, allowing for prolonged residence time at the site of action. The developed formulation showed better penetration, as estimated via confocal microscopy, and a more significant reduction in vascular endothelial growth factor levels in the retina after subconjunctival injection in a rat eye retinopathy model [143].

Polycaprolactone (PCL) stands out among biodegradable polymers due to its unique attributes, including biocompatibility, non-toxicity, controlled drug release, and gradual hydrolytic degradation [[144], [145], [146]]. It is a synthetic aliphatic polyester synthesized via ring-opening polymerization of epsilon-caprolactone, featuring a negatively charged carboxyl terminal group. The degradation rate of PCL varies depending on factors such as molecular weight, crystallinity, and environmental conditions, spanning from several months to 2-to 3 years. Additionally, PCL offers high drug permeability, making it a valuable component in drug delivery systems. PCL-based nanosystems have been developed for ocular drug delivery applications. For instance, PCL nanospheres and nanocapsules loaded with pilocarpine were developed for glaucoma treatment. Both systems exhibited minimal degradation over 42 days but displayed substantial degradation after 70 days in a pH 7.4 buffer solution. Notably, these nanocarriers effectively reduced intraocular pressure when injected into rabbit eyes [91,146,147]. PCL's hydrophobic nature results in rapid elimination from the body and nanoparticle instability in aqueous environments, necessitating surface modifications [91,146]. Researchers have in fact explored PEG-PCL polymeric micelles to enhance the water solubility of hydrophobic drugs with promising outcomes [145,148]. For instance, rapamycin encapsulated in PEG-PCL micelles administered via intravitreal injection in rabbits demonstrated extended retention in the retina, surpassing drug solutions [149]. Regarding safety, PCL is a biocompatible and non-toxic polymer used in FDA-approved pharmaceuticals. Evaluations confirmed the lack of irritation in the eyes and minimal cytotoxicity for PCL nanoparticles and PEG-PCL polymeric micelles, indicating their suitability for ocular administration. Various cytotoxicity tests revealed no substantial harm in eye-related cells within a broad concentration range [145,[150], [151], [152]].

#### 4.1.2 Conventional production methods

Conventional methods, known as *bulk mixing*, include a series of techniques leading to the precipitation of nanoparticles which may involve a prior emulsification of the polymer solution in an aqueous phase (solvent and surfactant) [[153], [154], [155]]. Nanoprecipitation, single emulsion (oil-in-water or water-in-oil), and double emulsion (water-in-oil-in-water) are among the most used techniques [25]. There are several factors to be considered before the production, including the solvent/surfactant choice, the solubility of the drugs, the mixing time for the aqueous and organic solvents, the concentration of polymer in the organic solution, and the ratio of organic to aqueous solution [156].

Nanoprecipitation relies on the interfacial deposition of a polymer from a lipophilic solution into an aqueous phase. In this technique, the polymer and drug are dissolved in a water-miscible organic solvent of intermediate polarity and added to an aqueous solution (which is not a solvent for the polymer) in a single step, stepwise, dropwise, or at a controlled rate, under stirring. As a result, the hydrophobic polymer precipitates, and forms nanoparticles by rapid diffusion of the polymer solution into the aqueous phase. This process necessitates a follow-up phase in which the organic solvent is removed either by reduced-pressure evaporation or by further stirring the solution [153,157]. Acetone is a commonly used water-miscible organic solvent easily removable through evaporation. The use of surfactants is not required for nanoparticle formation, although they can be used to ensure the stability of the colloidal suspension [153]. The physical and chemical properties of PNP can be controlled by adjusting the concentration and molecular weight of components, organic phase-to-aqueous phase ratio, organic phase injection rate, fluid dynamics, and mixing speed. According to Ref. [158], a low polymer concentration, a high injection rate, and a small needle gauge resulted in noticeably small PLGA nanoparticles, measuring 46 nm [112].

Emulsion methods can be classified as water-in-oil (W/O), oil-in-water (O/W), and double emulsions (W/O/W). The choice of emulsification technique relies on the features of both polymer and drug, as well as on the level of miscibility of the organic solvent with the water phase [25]. For instance, in the O/W approach, the polymer and drug are dissolved in a volatile organic solvent, such as dichloromethane or ethyl acetate, for a better toxicological profile [153]. The organic phase is then mixed into an aqueous phase containing a suitable surfactant, such as poly (vinyl alcohol) (PVA), under high shear stress (high-speed homogenization or ultrasonication [153]). The organic solvent is then allowed to evaporate (or diffuse, in the solvent diffusion variant [153]), which leads to the formation of nanoparticles [159]. The double emulsion (W/O/W) method, instead, is used to encapsulate hydrophilic drugs and proteins and involves two subsequent emulsifications [159,160]. The main parameters to control in these processes are polymer/surfactant concentration, solvent volume and water/oil ratio, and solvent/surfactant choice [153]. For instance, Kim. et al. addressed the emulsion-mediated production of latanoprost-loaded (a glaucoma medication) PLGA nanoparticles for their non-invasive (topical) permeation *in vivo* (iontophoretic-driven) [161]. In another work, Reis et al. developed sodium butyrate-loaded (NaBu-loaded) PLGA nanoparticles coated with chitosan (CS) for the treatment of choroidal neovascularization in wet-AMD models using a double emulsification and solvent evaporation technique [162].

Nanoprecipitation leads to the best results in terms of particle size distribution, reaching particles with diameters smaller than 100 nm with mild stirring (minimal shear stress). On the other hand, emulsion techniques can obtain higher drug encapsulation and loading efficiency. They however require an additional energy input (homogenization/sonication) [25]. Bulk mixing is a simple method but presents a series of limitations: it produces large nanoparticles with wide size distributions, which, depending on the application, are prone to poorer biodistribution, absorbance, and clearance [17], utilizing great quantities of polymers, solvents, and chemicals. This results in PNPs with high batch-to-batch variations, poor scale-up feasibility of the process, and hardly predictable physicochemical properties [154].

#### 4.1.3 Microfluidic production methods

A more adjustable, high throughput and reproducible method of PNP production involves microfluidics, which manipulates micro to picolitre volumes of fluids in channels with dimensions from tens to hundreds of micrometres [17,154,163,164]. Various materials like polymers, glass, or silicon can be used to produce microfluidic devices while techniques like soft lithography allow innovative device designs (i.e., hydrodynamic flow focusers (HFF)), which in turn enable the rapid and homogeneous mixing of reagents [17,25]. Microfluidics allows a precise control of reaction conditions, temperature, and reagent addition intervals. It also enables in-line characterization, feedback control, and high-throughput continuous synthesis for screening and optimizing NPs. This can lead to NPs with smaller dimensions and narrower size distribution, increased encapsulation efficiency, and slower release rates [17,165]. Since the typical Reynolds number is low (<100), the polymer-solvent mixing depends only on molecular diffusion, so appropriate micromixers are typically used to increase the mixing efficiency [166].

Passive mixing can be achieved using Y or T-shaped microfluidic devices with two inlets and one outlet, but it requires higher flow rates or obstructions to avoid wide distributions of residence time and particle size [154,167]. Two-dimensional HFF (2DHFF) methods are one of the most researched microfluidic NP production techniques. These methods involve directing a core stream of polymer dissolved in an organic solvent between two aqueous streams. The confinement of the polymeric solution shortens the diffusion length and mixing time (under 0.4 ms) and results in rapid self-assembly of NPs without solvent evaporation, improving nanoparticle uniformity [17,154,165]. A disadvantage of 2DHFF devices is that clusters of the polymer may develop at the interface between the central and lateral streams, causing device malfunction. To overcome this issue, 3D HFF devices have been designed, by eliminating the mentioned interface and preventing the device from clogging while producing nanoparticles with similar properties as 2D HFF devices [168]. Different configurations, such as parallelized channels [169] or embedded micromixers/micro-vortices [170], have been employed for polymeric nanoparticle formulations.

HFF devices require high flow rate ratios (focusing streams to central streams up to 20:1), limiting their suitability for scaled clinical production [165]. Alternative approaches like staggered herringbone micromixers (SHMs), flash nanoprecipitation, multi-inlet vortex mixers (MIVM), and confined impingement jet (CIJ) mixers have also been explored [[171], [172], [173], [174]]. Improved production methods, including microfluidic technologies, are crucial as therapeutic nanoparticles advance to clinical stages. Scalable microfluidic technologies are of growing interest, as single microfluidic devices typically produce micro-to-milligram quantities of NPs at rates below 10 mL/h, while clinical needs require gram-to-kilogram quantities or rates exceeding 10 L/h. Alternative designs with higher flow rates and pressures or patterned identical channels can enable a clinically more relevant production [17]. As an example, Streck et al. produced PLGA nanoparticles using a NanoAssemblr™ Benchtop Device from Precision NanoSystems (microchannels with dimensions of 200 μm width and 79 μm height). The microchannel featured a Y-shaped configuration with two inlets for the aqueous and organic solutions. It was designed with multiple layers to achieve a serpentine configuration and included an asymmetric in-floor herringbone groove at the bottom, which induced a transverse flow [175]. In another study, Morikawa et al. used a microfluidics system with a staggered herringbone structure to investigate the effects of experimental conditions on the characteristics of PLGA nanoparticles loaded with curcumin. They were able to generate small, uniform PLGA nanoparticles with high encapsulation efficiency and have verified that incorporating polyethylene glycol into the PLGA nanoparticles resulted in further size reduction and improved encapsulation efficiency while preventing the initial burst release of curcumin [176].

### 4.2 Characteristic PNP parameters

The determination of the administration route and drug delivery efficiency in ocular diseases, as well as subsequent therapeutic efficacy, are greatly influenced by the physicochemical properties of PNPs such as particle size, hydrophilicity/hydrophobicity, degradation characteristics and surface properties [42]. Therefore, it is important to focus on the physicochemical properties of nanocarriers that can lead to a more effective drug delivery for treating ocular diseases.

#### 4.2.1 Particle size

Polymeric nanoparticle size influences their ability to penetrate ocular barriers, the tolerance by the ocular district and the duration of drug action. Small nanoparticles may not provide an effective and sustained release, for their facile clearance to the blood and lymphatic stream but can surpass certain physiological barriers. On the contrary, larger PNPs, although impeded from reaching certain targets, may be useful for developing monthly-sustained drug release [42,[177], [178], [179]]. Practically speaking, PNPs with sizes between 50 and 400 nm are indicated as a versatile and effective product for ocular drug delivery, while particles exceeding 1 μm in size can potentially lead to irritation in the eyes [[180], [181], [182], [183]].

In the pre-corneal region, nanocarriers with sizes <200 nm can be easily absorbed by the cornea and conjunctiva through topical administration [184,185]. In the sclera, the interlacing and stacking structure of collagen fibrils constrains the transport of macromolecules. Hydrophilic nanoparticles with sizes ranging from 20 to 80 nm can diffuse towards the vitreous humour by passing through the sclera pores.

In the posterior segment, the movement of heavy therapeutic agents is hindered by the vitreous fluid. Particles with diameters smaller than 350 nm can reach the retina through intravitreal injection [186], while nanoparticles with a diameter inferior to 250 nm can evenly distribute in the vitreous and be absorbed by endocytosis from retinal cells [77]. Smaller objects (50 nm) have been shown to surpass retinal barriers and accumulate in the retina [183,187]. The drug load from particles as small as 20 nm was shown to rapidly deplete and be cleared to circulation in an *in vivo* subconjunctival retinal drug delivery model, while particles with a size between 200 and 2000 nm were able to remain in the delivery site for at least two months [178,179,186,188].

#### 4.2.2 Particle solubility

The solubility of PNP systems depends on the chemical composition, structure, and crystallinity of the constituting polymer and affects the drug release kinetics, stability and cellular internalization of the PNPs [189]. For instance, a higher molecular weight of the polymer increases its hydrophobicity, while greater branching in the polymer backbone enhances water solubility. On another note, drug release from PNPs obtained from hydrophobic polymers is mainly controlled by surface erosion [25,190] while the blending of hydrophilic polymers with hydrophobic polymers can lead to increased pore formation, faster polymer degradation, and accelerated drug release. For example, enhancing the hydrophilic glycolic acid portion of PLGA can result in faster degradation rates [25,191,192].

Solubility also affects the interactions of PNPs with different ocular structures [4]. Hydrophilicity enhances drug bioavailability in the tear film [193]. Hydrophilic particles can also pass more easily through the collagen-based pores of the scleral matrix but are hindered by the epithelium of the lens [42], and display enhanced cellular internalization in the sclera-choroid-RPE complex [194]. Hydrophobic substances, instead, cannot bypass the cornea through a transcellular pathway because its outer layer is hydrophobic, while the inner layer is hydrophilic [42]. Hydrophilic molecules can pass more easily through the collagen-based pores of the scleral matrix but are hindered by the epithelium of the lens [42]. The sclera-choroid-RPE barriers pose a more significant challenge for hydrophobic nanomaterials, while hydrophilic nanoparticles enhance cellular internalization [194].

Core-shell-based nanocarriers, with hydrophobic cores and hydrophilic shells, are interesting possibilities for the treatment of posterior segment ocular diseases since superficial hydrophobicity inhibits the muco-adhesiveness of nanoparticles, which is a fundamental property to increase the retention time between a nanocarrier and its biological target, thus ensuring a better absorption or intracellular uptake of a drug or the nanocarrier [112,[195], [196], [197], [198]].

#### 4.2.3 Particle degradability

Numerous studies have been conducted on degradable polymers to explore their potential as nanocarrier systems for ocular drug delivery. Degradable nanocarriers aim to enhance the therapeutic effects and at the same time improve the safety of ocular applications [107,112,183,196,199]. On the contrary, non-degradable nanocarriers, especially those based on polymers with high molecular weight, can accumulate in normal cells, leading to cytotoxicity with repeated use [25,139]. Degradable polymers commonly used in drug delivery contain labile chemical bonds, such as ester, anhydride, and amide, which can be broken down through hydrolysis or enzymatic cleavage. Degradable NPs offer several advantages in ocular drug delivery, including prolonged drug release, improved retention, and reduced cytotoxicity. The degradation rate of these NPs is of utmost importance for drug availability and efficacy. Several factors, such as composition, polymer crystallinity, glass transition temperature, solubility, molecular weight, and administration route, play a crucial role in the degradation and consequent drug release kinetics [4,25,[200], [201], [202], [203], [204]]. For instance, materials with low molecular weight degrade more rapidly, which results in a burst-type drug release and may compromise therapeutic outcomes. On the other hand, high-molecular-weight polymers may degrade too slowly, leading to material accumulation. Ultimately, the biodegradation mechanisms of nanomaterials and their outcome play a fundamental role in determining their clinical suitability for specific ocular applications [25,139,200,203].

#### 4.2.4 Particle surface charge and zeta potential

The surface charge of PNPs plays a crucial role in their drug delivery behaviour, influencing factors such as phagocytosis, penetration, and biodistribution [4,42]. Cationic nanoparticles, rather than anionic, have shown improved phagocytosis and cellular internalization. Moreover, electrostatic interactions based on surface charge enable targeted delivery to the retina through the cornea, even without the functionalization with specific ligands [145,205]. Positively charged nanocarriers face barriers in the lens and sclera, due to their interactions with negatively charged components, such as proteoglycans [4,206].

On the other hand, negatively charged NPs tend to avoid adhesion to healthy ocular surfaces and cells, prolonging their retention time. In the posterior eye segments, cationic NPs attract anionic components of the vitreous, leading to localized retention, while anionic particles diffuse more readily into the vitreous body [4,42,207]. It is important to consider that factors like stability and introduced ligands can impact the electrostatic behaviour of nanomaterials. Additionally, the size of nanoparticles can have a relevant influence on their charge if it exceeds 350 nm [207].

Zeta potential, the electrical potential difference measured at the boundary between the interfacial double layer of the dispersed particles and the solvent (continuous phase), known as a slipping plane, is a fundamental parameter of nano-formulations, that significantly impacts their stability and interaction with biological systems [208,209]. High zeta potential values (>±30 mV) lead to stable nano-formulations, thanks to the prevalence of electrostatic repulsion on attractive forces (van der Waals [209]). Since the electrostatic stabilization of nanoformulations with low zeta potential is limited by its kinetic nature and is applicable only to dilute systems with certain characteristics [209], nanoparticles with a zeta potential value ≤ 30 mV can be balanced via sterical stabilization (i.e., coating with polyethyleneglycol, known as PEGylation) [210,211].

#### 4.2.5 Surface modification of particles

Surface modification and engineering of nanoparticles have emerged as crucial strategies for enhancing ocular drug delivery by achieving thorough, controllable, and targeted release of therapeutic agents while mitigating their side effects. Overall, targeted approaches in ocular nanomedicine can be classified into passive and active strategies. Passive targeting relies on the physicochemical properties of NPs, such as size and charge, while active targeting involves the use of ligands to enhance cellular internalization in a specific manner [4,42,212]. Researchers have explored various surface engineering approaches in ocular nanomedicine [4,213,214].

The first approach involves decorating the surfaces of NPs with specific molecules or polymers to improve their physiological stability, bioavailability, and controlled drug release. For example, the enhancement of mucoadhesion through the functionalization of PNPs with hydrophilic cationic polymers such as PEG, chitosan, lecithin, and polymer-peptide moieties, improves adhesion, corneal uptake, and controlled release. These modifications have also shown promising results in enhancing ocular retention time, penetration, and therapeutic efficacy [111]. The functionalization with PEG, for instance, has been proven to prevent recognition and uptake by metabolic organs, as well as corneal uptake, overally improving drug bioavailability [[215], [216], [217]]. Ailing et al. developed dexamethasone-glycol chitosan nanoparticles to treat eye inflammation. The nanoparticles demonstrated mucoadhesive properties and were able to penetrate the corneal epithelium *in vivo*, making them a promising option for ocular drug delivery [218]. In another study, researchers developed chitosan-coated PLGA nanoparticles loaded with bevacizumab to treat posterior ocular diseases. The chitosan coating improved mucoadhesion to the sclera, resulting in extended residence time at the targeted site. The formulated mucoadhesive nanoparticles demonstrated enhanced penetration and reduced levels of vascular endothelial growth factor in the retina, indicating their potential efficacy in treating retinopathy when administered via subconjunctival injection [143].

A second approach focuses on the targeted modification with functional ligands/motifs to achieve site-specific therapy. This strategy relies on bioconjugation with affinity ligands such as peptides, proteins, and nucleic acids. By utilizing specific receptors expressed on ocular cells, targeted nanomedicine can deliver therapeutic agents to desired ocular tissues [219]. For instance, Alanazi et al. developed angiogenic suppressor–loaded PLGA nanoparticles decorated with internalizing arginyl glycyl aspartic acid (iRGD) peptide, capable of specifically targeting the ανβ3 integrin receptor, which is commonly overexpressed in the ocular vasculature of CNV-exhibiting patients. Human retinal microvascular endothelial cells (HRMEC) exhibited an enhanced uptake of the DDS when functionalized with iRGD, highlighting the potential of the functionalization [220]. Nguyen et al. [221] developed nanotherapeutics by aminolysis of resveratrol-encapsulated polycaprolactone nanoparticles (R@PCL NPs) and formation of amide linkages with carboxyl-terminated transactivator of transcription cell-penetrating peptide (T) and metformin (M). The resulting R@PCL-T/M NPs exhibited persistent in vitro drug release, biocompatibility, and potent bioactivity against key risk factors associated with retinal diseases. In vivo, a single intravitreal administration of R@PCL-T/M NPs significantly increased retinal permeability (approximately 15-fold), preserved endogenous antioxidants and inhibited abnormal vessel growth in the retina with macular degeneration for 56 days.

There are literature examples in which both strategies are employed to achieve interesting results. Wang et al. for instance, developed a dually functionalized DDS. The system consisted of a PEG-PLA-based nanoplatform decorated with a cell-penetrating peptide (CPP), which can be activated through a light stimulus and allows the accumulation of the PNPs in the neovascular lesions caused by AMD [96].

Another strategy involves embedding hardly deliverable therapeutic substances, such as nucleic acids (DNA, siRNA) within nanocarriers, allowing for their targeted release at pathological sites. This approach enables therapeutic functions where specific drug localization is essential. For instance, customizable nanocapsules based on covalently cross-linked polymers have been developed to deliver CRISPR-ribonucleoprotein (RNP) complexes for targeted gene editing [222].

Although surface engineering and mucoadhesion strategies hold promise for customized ocular medicine, it is important to make certain considerations. These include the selection of safe and non-toxic conjugated chemical moieties, maintaining functional chemical components, and ensuring the on-demand release of encapsulated substances, while preserving their therapeutic properties. In summary, surface modification and engineering of NPs, along with mucoadhesive strategies, have shown significant promise in overcoming the challenges of ocular drug delivery. These approaches enable the controllable and targeted release of therapeutic agents, while minimizing side effects, paving the way for personalized ocular medicine in specific paradigms [42,80,212].

### 4.3 Drug release behaviour and nanocarrier fate

Diffusion and polymer erosion are the main dynamics that govern drug transport out of PNPs. While diffusion is driven by chemical gradients or osmotic pressure-generated convection, erosion leads to pore formation and its effects become evident after an initial diffusion-controlled step [25,212]. Drug release behaviour plays a fundamental role in the design of PNPs and is closely related to stability, therapeutic outcomes, and formulation development [212,223]. The main controlled-release mechanisms [224] can be summarised as:

• **Drug diffusion through water-filled pores**: in degradable polymeric systems drug molecules move randomly through pores, controlled by concentration gradients. Water absorption by PNPs is faster than drug release. Pores enlarge over time, facilitating the release [224].
• **Diffusion through the polymer matrix**: in nondegradable systems drug molecules diffuse out of the polymer matrix at a constant rate, in fact, diffusion only depends on membrane properties such as permeability and thickness [225].
• **Osmotic pumping**: convection-driven drug transport through water-filled pores, caused by osmotic pressure [226].
• **Erosion**: divided into *surface erosion*, a controlled and reproducible degradation of the matrix/scaffold from the outside-in (protects water-sensitive drugs), and *bulk erosion*, in which water permeates the bulk, resulting in uniform matrix degradation (less predictable, no protection for drugs from the environment) [227].

It is clear how understanding polymer matrix degradation is crucial for controlling drug release kinetics in DDSs [228]. In fact, adjusting the polymer degradation rate fine-tunes active substance release kinetics [229]. When polymers are used to fabricate DDSs, degradation may occur via hydrolytic (water-labile bonds scission) or enzymatic (e.g., via hydrolases or proteases) mechanisms [203,230]. In the first case, degradation may be controlled, for example, by varying the hydrophilicity of the polymer (adding conjugate structures, side groups, or varying the dielectric constant) [203]. For instance, PLGA hydrolytic degradation can be varied by adjusting the copolymeric ratio of the glycolic and lactic monomers.

On the other hand, enzymatic degradation can be influenced by the interactions between the enzyme and the polymeric chain (diffusion or adsorption), the physicochemical properties of substrates (i.e., molecular weight), environmental conditions (i.e., pH and temperature), and the presence of activators or inhibitors in the surrounding medium [203]. A study by El-Sherbiny has highlighted how the enzymatic hydrolysis of chitosan nanoparticles using lysozyme, a 14 kDa cationic protein, can be reduced by grafting chitosan with PEG [231]. Moreover, mesh size affects drug diffusion. If mesh size surpasses drug size, diffusion dominates; comparable sizes cause steric hindrance unless the pores are enlarged by degradation, swelling, or external forces; increasing cross-linking degree or polymer concentration reduces mesh size, slowing drug diffusion for extended-release [230]. To summarize, biodegradation depends on polymer properties like structure, weight, hydrophilicity/hydrophobicity, unstable bonds, morphology, and copolymer ratio. These properties can be manipulated to extend formulation effects [203,230].

Polymeric DDSs commonly exhibit triphasic drug release profiles, characterized by an initial burst release, due to the release of drug molecules close to the surface of the particles and therefore near the water layer. Burst release can be triggered by osmotic pressures, rapid environmental changes, or other factors. Strategies to minimize burst release include coating the system, using high-molecular-weight polymers, achieving nonuniform drug distribution, and decreasing polymer hydrophilicity [232]. This first burst phase is then followed by a slow controlled-release phase, mainly governed by diffusion and partially by erosion dynamics. Finally, the last (and generally faster than the previous one) release phase is governed by erosion. Nonetheless, the release behaviour depends on various factors and should be studied on a case-by-case basis [224].

*In vitro* investigations of drug release profiles need to be supplemented with real-time monitoring methods for accurate evaluation *in vivo* [233]. Mathematical modelling may also play a crucial role in predicting and optimizing drug release from delivery systems. Several mathematical models are commonly employed in drug release studies, including the zero-order kinetic, first-order kinetic, Higuchi matrix, Korsemeyer-Peppas, and Hixson Crowell models [234]. Further advancements in modelling techniques are needed to capture the intricacies of multi-component systems and responsive drug release mechanisms [225].

Finally, it is worth mentioning that the eye is an organ with independent metabolic activity as it contains various metabolic enzymes distributed throughout its tissues, which are involved in the breakdown of nanocarriers. The role that ocular metabolism plays in drug delivery has been deeply investigated [235]. For instance, PLA and PLGA undergo hydrolysis, while some polysaccharides degrade via enzymatic hydrolysis and oxidative fragmentation. Inorganic-based nanocarriers can undergo enzymatic and non-enzymatic degradation. Clearance of inorganic nanoparticles occurs through cellular internalization. Unmetabolized materials and metabolites circulate systemically and can be cleared by the spleen, liver metabolism, and kidney filtration. Strategies to improve tissue targeting and reduce systemic distribution involve controlling nanocarrier size and surface chemistry [236]. A detailed analysis of nanocarrier absorption, distribution, metabolism, and excretion of nanocarriers was performed by Su et al. [237]. Ultimately, controlled-release devices using degradable polymers are of high interest for their capability of providing temporal and spatial control over drug release. Other aspects which must be taken into consideration while designing these products are the optimal dosing of the nanocarriers to obtain the desired effects without hindering patient compliance and minimizing underexposure and toxicity risks [25].

## 5 Light responsive nanoparticles

Stimuli-responsive polymers have gained growing interest thanks to their property-changing response to many different variables (temperature, pH, ions, enzymes, and light) [[24], [25], [26]]. With specific reference to ocular drug release, light-responsive materials are particularly interesting, as the eye allows the penetration of various light wavelengths, thanks to the transparency of the cornea and lens, potentially surpassing limitations of endogenous stimuli like pH, temperature, and ions and [25,[30], [31], [32], [33]].

The crucial requirement for light activation of polymeric nanocarriers is the inclusion of specific additives able to harness light energy. These can serve as photosensitizers, possess photoreactive chemical groups, or function as key constituents involved in various photoinduced mechanisms associated with DDSs [238]. The irradiation of photoactive materials can induce various photophysical effects, including reversible (such as isomerization and cleavage) and irreversible (such as dimerization/polymerization and photosensitization) changes, along with photothermal effects [39].

**Fig. 4:**
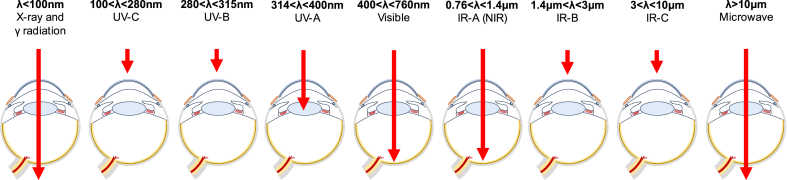
Schematic of the different penetration depths for light sources into the eye spanning various wavelengths.

Light-responsive DDSs demonstrate precise spatiotemporal controllability, flexibility in wavelength selection (Fig. 4), minimal side-product generation, convenience, and ease of use. They can be used without direct contact, eliminating the need for additional reagents. The rate of drug release can be optimized by adjusting the wavelength, intensity, and duration of the light irradiation. This level of control can enable tailored drug delivery applications, which can in turn lead to the reduction of the number of administrations necessary to reach effective dosages of a drug and allow the minimally invasive delivery to the posterior eye segment [1,29,39].

It is important to note that light above 900 nm is phototoxic for retinal tissues. Additionally, due to ocular surface absorption, wavelengths between 280 and 315 nm (UV–B) and 100–280 nm (UV–C) can't fully penetrate the cornea. Thus, caution is advised when using light in these ranges to trigger ocular light-responsive DDSs [30]. Light-sensitive DDSs for ocular drug delivery have been designed employing ultraviolet (UV, 200–400 nm), visible (Vis, 400–700 nm), and near-infrared (NIR, 700–1000 nm) wavelengths as light sources. These systems enable controlled release of the drug through on/off sequences or complete release upon activation [39,239]. Further elaboration on these systems will be provided in the following discussion.

### 5.1 UV–vis light controlled drug delivery systems

UV–vis light-responsive controlled release involves the use of specific compounds in which a transformation is induced following the exposure to UV–vis light. There are three main ways in which UV–vis light may control an ocular drug release dynamic:

• **Photocleavage**: photolabile groups, such as *o*-nitrobenzyl (oNB) or organic compounds, such as coumarins, may be incorporated in the backbone of polymers or used to bridge the link between a polymer and a drug and undergo a light-induced bond-cleavage [32,240].
• **Photosensitization**: photosensitizers are organic (i.e., indocyanine green, hypocrellin B) or inorganic (i.e., TiO2) molecules that absorb light and transfer its energy to neighbouring molecules through energy or electron transfer leading to the production of reactive oxygen species (i.e., singlet oxygen, hydrogen peroxide) upon illumination of the sensitizing molecule while molecular oxygen is present. This technique is utilized in photodynamic cancer or neovascularization therapy, where the reactive oxygen species generated by a photosensitizer destroy target tissues or cells through apoptosis [56,241,242].
• **Photothermal effects**: photothermal reactions employ a plasmonic material, light activation, and a thermally sensitive drug carrier. Gold (Au) nanoparticles for instance rapidly absorb energy from UV, visible, and NIR sources, releasing it as heat. When matched with the nanoparticles' absorption bands, the generated heat can cause carrier rupture and payload release. Au nanoparticles are preferred for their biocompatibility, and customizable optical and photothermal properties [56,243].

**Fig. 5:**
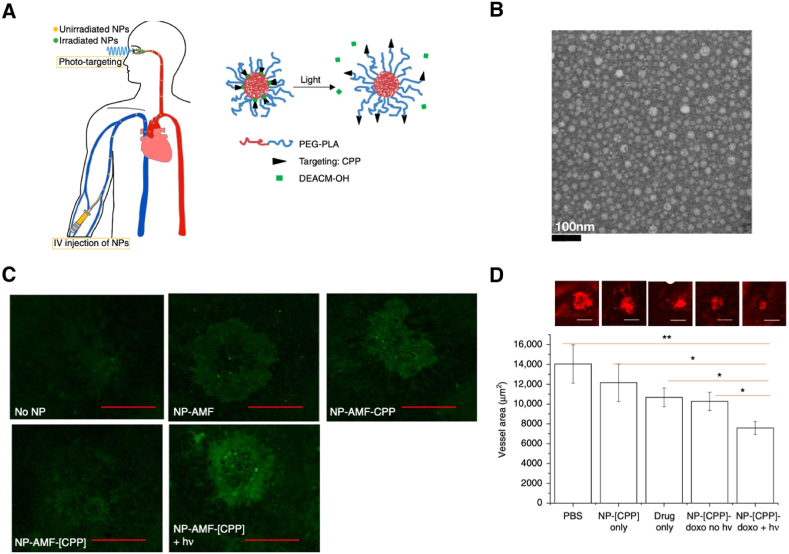
A novel photocleavable PNP formulation for the treatment of CNV devised by Wang et al. [96]. A) A scheme of the phototargeting strategy adopted by the researchers. The NP-[CPP], composed of maleimide-modified PEG-PLA, the CPP and the photocleavable group DEACM-OH are injected intravenously and activated by a blue-light radiation directed at the eye. B) Transmission electron microscopy (TEM) image of the NP-[CPP] complex obtained by thin film hydration. C) In vivo phototargeting of a CNV model. Shown here is one representative image (out of 8) of fluorescently labelled flat-mounted choroid tissues captured 24 h post-injection with nanoparticles (NPs). The scale bar corresponds to 100 μm. D) Exemplary images of choroidal neovascularization (CNV) stained with isolectin GS IB4 (scale bar: 100 μm) and a histogram of the average CNV lesion size. These images depict CNV in laser-induced mice treated with different modalities: PBS, NP-[CPP], doxorubicin (doxo), NP-[CPP]-doxorubicin, and NP-[CPP]-doxorubicin with irradiation. The error bars represent the standard error of the mean, and the dataset comprises a total of 28 CNV lesions. Statistical significance is denoted as follows: *P < 0.05, **P < 0.005, determined using an unpaired *t*-test. Adapted under the terms of the Creative Commons CC BY license from Wang et al., 2019, Ref. [96]. (For interpretation of the references to colour in this figure legend, the reader is referred to the Web version of this article.)

In their study, Wang et [96] al have devised a novel formulation of nanoparticles denoted as NP-[CPP] (Fig. 5). These nanoparticles are designed for the treatment of CNV through intravenous administration through light-responsive targeting mechanisms (Fig. 5, A). NP-[CPP] were created using the thin-film hydration method, obtaining a hydrodynamic diameter of 19.0 ± 2 nm (Fig. 5, B). The construction of NP-[CPP] entails the modification of maleimide-modified PEG-PLA chains with a cell-penetrating peptide (CPP). Notably, the attachment of a DEACM photolabile moiety to the CPP effectively hinders the cellular internalization of NP-[CPP]. Subsequent exposure to blue light serves to cleave the DEACM component from the CPP, enabling the CPP to translocate from the nanoparticle core to its surface, thereby activating its functional properties. Notably, the NP-[CPP]+hν group displayed a greater fluorescence intensity in the eyes of mice compared to the other groups (NP, NP-CPP, and NP-[CPP] without irradiation), thereby confirming the efficacy of photo-targeting in enhancing nanoparticle accumulation within the CNV areas (Fig. 5, C). These nanoparticles have shown remarkable efficacy in a murine model of CNV. When loaded with doxorubicin, the irradiated NP-[CPP] formulation demonstrated a significant reduction in neovascular lesion size compared to control groups. Specifically, mice treated with NP-[CPP]-doxorubicin and irradiation exhibited a 46.1% reduction in neovessel area, surpassing the outcomes observed in the free doxorubicin group and the NP-[CPP]-doxorubicin without irradiation group, which displayed reductions of 24.0% and 26.8%, respectively (Fig. 5, D). These findings underscore the potential of NP-[CPP] as a promising strategy for enhanced and localized drug delivery in the context of CNV management.

For the photodynamic treatment of AMD, Ideta et al. successfully loaded a dendritic porphyrin into polyethylene glycol-block-poly (l-lysine) nanocarriers to enhance its therapeutic effects. The unique dendritic structure of the porphyrin prevented core sensitizer aggregation, leading to a highly efficient photochemical reaction. The loaded micelles demonstrated superior accumulation in diseased lesions compared to free dendritic porphyrin, resulting in highly effective occlusion of choroidal neovascularization (CNV) with minimal phototoxicity. Additionally, light-induced oxidation was utilized to achieve a controlled release of therapeutic agents from oxidizable nanocarriers/endosomes by irradiating a photosensitizer, causing disruption of carrier/endosome membranes [244].

Basuki et al. took advantage of the photothermal conversion operated by Au NPs under UV–vis irradiation to develop a light-triggered hydrogel for controlled delivery of bevacizumab, aimed at treating AMD. The hydrogel consisted of agarose embedded with poly (METAC) functionalized AuNPs and demonstrated the ability to release the drug upon irradiation with visible light (400–500 nm) for successive cycles. This light-triggered release system significantly enhanced drug release compared to hydrogels without nanoparticles, achieving a three-fold increase. The release profile could be adjusted by varying the concentrations of AuNPs and agarose and the intensity and duration of light exposure. The released bevacizumab demonstrated efficient inhibition of human vascular endothelial growth factor activity, even at lower concentrations [243].

While various materials and additives can respond to UV radiation, the medical applications of UV-light-responsive nanocarriers are limited due to the high energy of UV radiation, which can be harmful to human tissues, and their limited tissue penetration [32,56]. Groups photosensitive to higher wavelength radiation, such as near-infrared (NIR), have thus been developed [25,245,246].

### 5.2 NIR light controlled drug delivery systems

Near-infrared (NIR) radiation possesses high tissue penetration depth due to its low absorption by endogenous chromophores (i.e., blood, water, and melanin) and biomolecules, leading to minimal damage to cells. There are two main ways in which a NIR-responsive molecule may favour drug delivery:

• **Photo-oxidation**: by irradiating photosensitizers with the appropriate wavelength light, reactive oxygen species (ROS) can be generated through a photodynamic effect and disrupt biomembranes of nanocarriers (i.e., lipid nanoparticles, lipid bilayers [247]) or cleave specific bonds (i.e., oxidizable diselenide bonds [248]) leading to the release of the loaded pharmaceutical cargo. These photosensitizers often have tetrapyrrole structures (i.e., phthalocyanines, porphyrins) or are certain dyes (i.e., boron dipyrromethene) or natural products (i.e., curcumin). An extensive review of these molecules can be found in Refs. [238,249]. For instance, a poly (methacrylic acid)-based nanogel, equipped with a ROS-generating indocyanine green dye, demonstrated NIR light-responsive drug release by photocleavage of a diselenide linker through ROS generation [248].
• **Photothermal effect:** photothermally triggered drug release involves converting absorbed light energy into heat, which then triggers cargo release from heat-sensitive nano vehicles. Noble metal NPs, such as gold [250], can act as photothermal converting agents due to their excellent optoelectronic properties (through their characteristic localized surface plasmon resonance (LSPR) [238]). Additionally, nano carbons [251], polymers such as PDOPA [129,133], and specific dyes, such as the cyanine-based and FDA-approved ICG [31] and IR820 [252], can exhibit photothermal activity. By employing NIR with appropriate power densities, the viability of tissues and cells can be preserved [238].

**Fig. 6:**
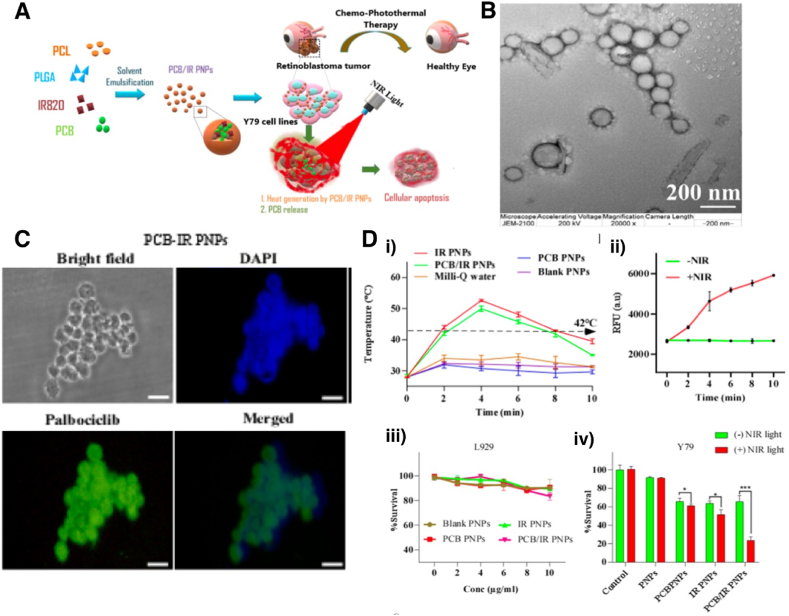
The multifunctional PNP DDS designed by Mudigunda et al. [252]. A) A scheme depicting the design principle of the DDS, composed of PCL, PLGA, the NIR-photoresponsive dye IR820 and the drug PCB. B) A TEM image of the produced PNPs, sizing between 170 and 200 nm. C) Fluorescence microscopy images of the PCB/IR PNP-treated Y79 retinoblastoma cell line, illustrating the internalization capabilities of the DDS. D) Functional behaviour of the DDS i) Photoresponsive behaviour of the PNPs in three different formulations, incorporating either only the IR820 dye, only the PCB drug, neither of them or both, plotted against milli-Q water as control. ii) The time-dependent PCB release under NIR irradiation as opposed to the non-irradiated sample. iii) A graphic visualization of the biocompatibility, in terms of % of survived cells, of various concentrations of the PNPs, as tested on an L929 cell line using the four different formulations described in bullet point D-i. iv) The cytotoxic effect on a retinoblastoma cell line of the four different PNP formulations either under NIR-irradiation or not. The data are plotted as mean ± standard deviation. The variations among groups were considered significant for *P < 0.05, **P < 0.01, and ***P < 0.001. Adapted with permission from Mudigunda et al., 2022. Copyright 2023 American Chemical Society, Ref. [252].

The photothermal effect has been employed in some recent applications in the field of PNP-mediated ocular drug delivery. For instance, Mudigunda et al. have designed multifunctional PLGA-PCL nanoparticles (Fig. 6) loaded with the FDA-approved anticancer drug Palbociclib (PCB) and the near-infrared dye IR820 (IR). These PCB/IR PNPs served as chemo/photothermal agents for retinoblastoma therapy (Fig. 6, A). The PNPs were synthesized through the solvent emulsification method. Examination via electron microscopy techniques (TEM, AFM, and FE-SEM) revealed that the PCB/IR PNPs exhibited a uniform and spherical morphology within a size range of 170–200 nm (Fig. 6, B). The assessment of the PNPs' cellular uptake was conducted on Y79 cells using a fluorescence microscope. After a 5-h incubation, PCB and PCB/IR PNPs exhibited conspicuous green fluorescence within the cytoplasm, while the respective control group displayed no fluorescence signal (Fig. 6, C). By assessing the photothermal effect, it was observed that PCB/IR PNPs exhibited a time-dependent increase in temperature when subjected to near-infrared (NIR) light (Fig. 6D–i), which facilitates the controlled release of PCB, thereby yielding a sustained impact on tumour cells and the disintegration of the PNP shell, which enables the release of PCB. For the evaluation of the triggered release profile of Palbociclib (PCB) from PCB/IR PNPs, fluorescence measurements were taken before and after 780 nm laser irradiation and the data were plotted as % PCB release (Fig. 6D–ii). Biocompatibility studies were conducted on L929 cell lines, revealing that all types of PNPs (PCB, IR, and PCB/IR) were compatible with normal L929 cells at concentrations up to 10 μg/mL (Fig. 6D–iii). *In vitro* evaluations of the combined chemo/photothermal effect of the PNP formulation using a retinoblastoma (Y79) cell line demonstrated significant cytotoxicity (86.5 ± 2.3%) of the PCB/IR PNPs upon NIR light exposure compared to control groups (Fig. 6D–iv). Further analyses revealed that DNA damage and subsequent apoptosis were induced by the PCB/IR PNPs in the presence of NIR light. *In vivo* studies also showed a potential for photoacoustic imaging through the nanocarriers allowing the detection of the PCB/IR PNPs via an MX-400 30 MHz linear array ultrasound transducer [252].

In another study, Tao et al. designed a nanocomposite thermogel composed of hexanoyl glycol chitosan (HGC) and mesoporous poly-dopamine nanoparticles (MPDOPA NPs), which exhibited a reversible sol-gel transition, injectability, and *in situ* gelation capability. By incorporating MPDOPA NPs, the thermogel displayed excellent photothermal conversion ability, leading to efficient temperature elevation upon 808 nm laser irradiation. This feature facilitated a controlled drug release profile that could be regulated by NIR laser activation. *In vitro* studies confirmed that Hexanoyl Glycol Chitosan embedded with Mesoporous PDOPA nanoparticles loaded with Doxorubicin (HGC-MPDOPA@DOX), when exposed to NIR laser irradiation, exhibited a superior anticancer effect on OCM-1 cancer cells compared to any single treatment approach, demonstrating the potential of the HGC-MPDOPA thermogel as a versatile platform for intraocular tumour treatment [253].

Table 1 summarises some recent applications of UV–vis/NIR-triggered release from PNPs.

**Table 1:** Summary of recent applications of light-triggered drug release from PNPs.

Light source	Nanocarrier	Drug	Fabrication	Release mechanism	*In vivo* model	Application	Highlights	Further studies	Case
UV (365 nm)	A novel polymer containing multiple light- sensitive groups and incorporating a quinone-methide self-immolative moiety	*Nintedanib (BIBF 1120)*	O/W emulsion	*Photocleavage of the oNB group in a quinone-methide system*	*Light-triggered release of Nintedanib in a murine model*	*Designing an on-demand DDS for the treatment of AMD*	- brief, low-power-light exposure - *in vivo* release up to 30 weeks post-injection - suppression of CNV *in vivo* up to 10 weeks post injection	- In vitro release kinetics and modeling - Release of multiple dosages under examination	**Huu et al 2015** [33,254]
**UV**–Vis (400 nm)	mPEG-PLA NPs modified with a cell penetrating peptide (CPP) and 7-(diethylamino)-4-(hydroxymethyl) coumarin (DEACM) photocleavable group	*Doxorubicin*	Self-assembly under stirring	*Photocleavage of DEACM*	*Light-triggered targeting of the formulation in a murine model*	*Intravenously administered nanoparticles for CNV treatment*	- surface modified, CPP-functionalized and intravenously administrable nanoparticles, with low-power-light - triggered targeting - NP accumulation in neovascular lesions and significant reduction of lesion size *in vivo* (comparable to locally administered drugs)	- changing the drug used - modifying drug dosage and irradiation parameters - disease model does not represent the chronicity of wet-AMD - NP formulation improvements - retention and clearance of doxorubicin in mouse organs not monitored	**Wang et al 2019** [255]
Vis (420 nm)	Self- assembled dicyanomethylene-modified, trigonal core molecule (TAEA) - conjugated DEACM nanoparticles. NPs pegylated with DSPE-mPEG	*Doxorubicin*	One-step nanoprecipitation	*Photocleavage of DEACM*	*Photo-triggered DOX release in orthotopic retinoblastoma- bearing mice*	*Photoresponsive intravenous drug release system for the treatment of retinoblastoma*	- low-power-light -triggered drug accumulation *in vivo* and significant anti-cancer effect - low side effects/non-specific delivery	- light-responsive drug delivery system can be applied to other diseases/organs	**Long et al 2021** [256]
**UV**–Vis (400–500 nm)	Poly (METAC) functionalized gold nanoparticles (embedded in agarose hydrogels)	*Bevazucimab/Avastin*	Reduction of gold chloride with sodium citrate in aqueous solution and addition of thiol-ended poly (METAC)	*Photo-thermal conversion (gold NPs)*	*-*	*On demand DDs for the anti-VEGF treatment of AMD*	- low-power-light - triggered photothermal conversion leads to controlled burst-type drug release from agarose depot - biological activity of bevacizumab highly retained (maintained over therapeutic level)	- the delivery system will be studied for its *in vivo* ocular bioavailability and biocompatibility	**Basuki et al 2017** [243]
Vis (532 nm)	Fucoidan - gold nanoparticles	*Doxorubicin*	Self-assembly under stirring	*Photo-thermal conversion (gold NPs)*	*Photo/chemothermal efficacy assessed on VX2 tumor-bearing rabbits*	*Dual photothermal therapy (PTT) and chemotherapy of eye tumours*	- high cytotoxicity on the tumor cells and strong light absorption in vitro - NPs lead to the complete removal of tumours without recurrence for 14 days - Enhanced photoacoustic image contrast from the tumor regions	- potential theranostic formulation for treating and diagnosing ocular tumours	**Kim et al 2017** [257]
Vis (590 nm)	PLGA-TPGS copolymeric nanoparticle loaded with nanosilver for singlet oxygen generation enhancement and hypocrellin B photosensitizer	*-*	Nanoprecipitation	*Photodynamic ROS generation mediated by hypocrellin B (HB) and nano-silver*	*In vivo biodistribution of NPs to evaluate uptake of photosensitizer by ocular tissues*	*CNV blocking and excess blood vessel eradicating nanosystem for the treatment of AMD*	- effective phototoxic effect with 2 h irradiation observed in A549 cells - significant anti-angiogenic effect on CAM embryos - ROS generating capability enhanced by addition of nanosilver - optimal uptake, biodistribution and retention of photosensitizer HB by ocular tissues	- proof of concept study which may be extended to design an efficient photodynamic therapy formulation	**Krishnaswami et al 2018** [242]
Vis (670 nm)	Perfluorocarbons and pyropheophorbide (Ppa) anchored acetylated-HA nanoparticles	*-*	Nanoprecipitation	*Photodynamic ROS generation mediated by Ppa photosensitizer*	*OM431 cells injected in BALB/c nu/nu mice, injection of nanoformulation and irradiation of mice 10 days post tumor transfection*	*Novel DDs for the treatment of ocular choroidal melanoma by photo dynamic ameliorating hypoxia*	- Self-carrying photodynamic therapy system - higher singlet oxygen yield thanks to perfluorocarbons - targeting capability thanks to HA moiety, which leads to improved cellular uptake	- optimization of the nanoformulation needed before clinical application can be considered	**Li et al 2020** [258]
NIR (780 nm)	IR820 loaded PLGA-PCL NPs	*Palbociclib*	O/W emulsion	*Photothermal conversion (IR820)*	*In vivo effects assessed by administering PNPs to female Balb/C mice and observed for 14 consecutive days. Blood and organs were collected*	*Dual PTT and chemotherapy of retinoblastoma*	- temperature increase within minutes of irradiation - excellent PCB bioavailability in retinoblastoma cells - remarkable amount of cell death (86.5 ± 2.3%) due to the chemo/photothermal combinatory effect	- potential as a cost-effective and non invasive treatment of retinoblastoma for future preclinical studies	**Mudigunda et al 2022** [252]
NIR (808 nm)	IR820 loaded PCL NPs	*Biomolecular fraction of Moringa oleifera (DFM)*	O/W emulsion	*Photothermal conversion (IR820)*	*-*	*Dual PTT and chemotherapy of retinoblastoma*	- PTT combined with PNPs demonstrate synergistic effects - PNPs downregulate the expression of HSP70, diminishing the heat shock resistance of tumour cells	- further studies such as flow cytometry, Western blot, or qRT-PCR for the evaluation of expression of apoptotic/autophagic markers to define the role of DFM/IR820 mediating PTT in the synergistic cell death - *in vivo* assessment	**Mudigunda et al 2023** [259]
NIR (785 nm and 808 nm)	Poly (styrene sulfonic acid) sodium salt (PSS) decorated, ICG loaded poly (allylamine hydrochloride) (PAH) microparticles	*Avastin*	Co-precipitation technique	*Photothermal conversion (ICG)*	*-*	*On-demand, low frequency treatment of DR*	- non-toxic internalization in human retinal pigmented epithelial cells - increased cell viability following irradiation and avastin release	- *in vivo* assessment, especially regarding ocular tolerance of microcapsules	**Stoia et al 2023** [260]
NIR (808 nm)	Mesoporous PDOPA nanoparticles	*Doxorubicin*	Template removal – self-assembly method	*Photothermal conversion (PDA)*	*-*	*Chemo-photothermal treatment of intraocular cancer (uveal melanoma, retinoblastoma)*	- significant photothermal capabilities of PDA NPs, unaffected by incorporation in thermogel for drug depot applications - sustained release of drug enhanced by ∼25% following NIR irradiation - survival of OCM1 cancer cells below 25% following NIR irradiation and drug release	- optimization of irradiation-linked release effects - *in vivo* assessment	**Tao et al 2023** [253]

### 5.3 Limitations of light-triggered release

Two of the main challenges in the development of light-responsive biomaterials for ocular drug delivery are ocular light transmittance and the safety of the light source. Light transmittance in the eye is wavelength-dependent [39]. Although UV radiation has limited ocular transmittance, UV-A (315–400 nm) can penetrate the entire lens and reach the retina. However, as already mentioned, UVs are associated with cytotoxicity [261]. Near-infrared (NIR) radiation (760–1400 nm) offers superior tissue penetration and lower absorption by water and lipids. The wavelength, intensity, and irradiation time of light determines the type of tissue damage, which can be thermal, photomechanical, or photochemical. Photothermal and photomechanical damages are associated with NIR exposure, while photochemical effects primarily occur with UV light exposure [261]. Generally, longer wavelengths have a higher safety profile due to their lower energy, making NIR irradiation preferable to UV light for ocular applications [39,261,262]. Nonetheless, NIR radiation presents its set of limitations, being unable to prompt fast reactions due to its low energy, which may result in a burst release of drugs loaded in a nanocarrier [56]. Moreover, few compounds directly respond to NIR light or can sense the low energies imparted [238]. To overcome this, compounds that convert NIR light to UV and/or Vis light have been developed by exploiting nonlinear photoconversion mechanisms, such as two-photon absorption (TPA), hot band absorption (HBA) and upconversion (UC) [32,56]. These processes rely on the properties of anti-Stokes luminescent materials to produce high-energy photons from the excitation operated by low-energy photons (in other words, from short to long-wavelength photons). Additional energy is needed for the conversion to operate, such as heat or additional photons. Anti-stokes conversions allow a user to operate with, for example, NIR-range exciting photons, maintaining their high penetration depth, while still exploiting the high energy of the resultant short wavelength photons (i.e., in the UV range) [263].

**Fig. 7:**
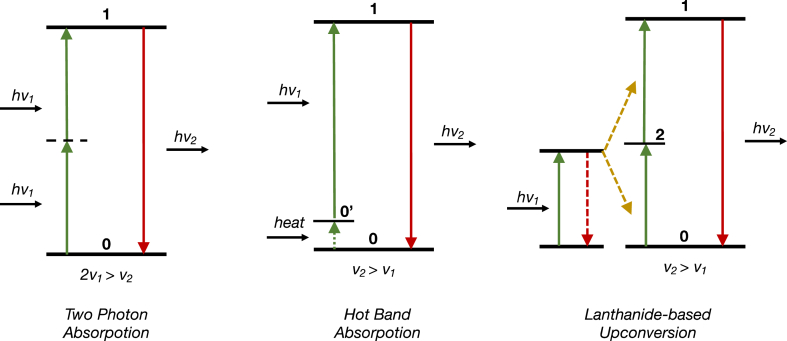
A schematic of the functioning principles of the cited nonlinear photo conversion processes, as detailed in paragraph 5.3.

TPA involves the simultaneous absorption of two photons, propelling a molecule from the ground state (0) to a higher energy level (1) (Fig. 7). The returning photons from the state (1) emit a higher energy photon than the absorbed ones, equal to the sum of the initial energies. TPA involves the use of a TPA-sensitive fluorophore and a sensitizer. The fluorophore molecule absorbs two photons and emits fluorescence which activates the photosensitizer, which is either covalently bound to a nanomaterial or doped within it. Finally, the drug release takes place either by photocleavage, the dynamic of which has been displayed in the precedent paragraph, or photoisomerization [238,264]. Photoisomerization is a reversible change in molecular conformation induced by UV or visible light involving compounds transitioning between trans and cis conformations with a restricted and rotation-induced transformation of a double bond. These systems act as valves, enabling an on/off control of drug release. Spiropyrans and azobenzene groups are examples of compounds which undergo photoisomerization when exposed to UV light [32,39,239]. Limited research has been conducted on the application of these functional groups in the field of ocular DDSs [39]. TPA dyes necessitate a high energy-density pulsed laser to induce emission, which can lead to photobleaching and heating concerns [32,238,263,265].

In HBA, additional energy is provided by the heat from the initial distribution of fluorescent dye molecules (Boltzmann distribution, Fig. 7). High-vibration energy level molecules, known as the ‘hot band,’ can absorb lower energy photons and transition to an excited state (1). The excited state then returns to the ground state, resulting in shorter-wavelength fluorescence emission (1–0). Limited research exists in this field due to a lack of clear design guidelines for efficient dye molecules. Current hot band absorption materials mainly cover the longer-wavelength portion of the visible region. Commercially available dye molecules such as Fluorescein, Rhodamine 101 or B, and Oxazyne 1 have been reported to demonstrate high conversion efficiency [263,266].

Upconversion generates anti-Stokes luminescence using multiple intermediate states to accumulate low-energy excitation photons (Fig. 7). Real intermediate states increase the probability of photon absorption in UC compared to TPA. Two main upconversion mechanisms exist. The first uses trivalent lanthanide ions (e.g., Yb3+, Nd3+, Er3+ or Tm+). The second process is the triplet-triplet annihilation (TTA)-based upconversion, which exploits a sensitizer and an emitter with a matching triplet state [32,263,267]. Upconversion offers several advantages such as preventing overheating issues and minimizing photodamage to tissues and cells, while allowing the use of continuous-wave lasers. Additionally, it enables deep tissue penetration, enhances detection sensitivity (i.e., NIR light as low as 0.35 W*cm^−2^ [268]), reduces autofluorescence, and provides a high signal-to-noise ratio [238]. Similarly, as for TPA, upconverted photons can sensitize photoactive molecules (encapsulated, self-assembled, covalently bound or not, or adsorbed to the surface of nanoparticles [268]) via photoisomerization or photocleavage and deliver the drug cargo [238]. Despite the impressive research conducted on upconversion nanoparticles (UCNPs), there are concerns regarding their potential long-term *in vivo* toxicity, due to the presence of non-biodegradable metals [269,270].

The TPA, HBA and UC processes described differ in terms of luminescence lifetimes (longer for upconversion processes) and linearity of the curve of emission intensity (non-linear for upconversion processes). A detailed analysis of these mechanisms is portrayed in Ref. [263].

## 6 Conclusions

Polymeric nanoparticles (PNPs) have emerged as versatile tools for drug delivery in ophthalmology, offering potential solutions to conventional therapy challenges [39,56]. In particular, polyesters, such as PLGA and PCL, are commonly chosen for their excellent biocompatibility and biodegradability. Although they pose challenges such as protein instability and inflammation response, efforts to optimize PNPs through surface modifications and incorporation of biologically responsive components for controlled drug release are meaningful strategies to obtain a more precise and efficient delivery [42,271]. These design modifications enable improved drug delivery to the eye by enhancing retention time, facilitating drug penetration through barriers, and targeting specific cells and tissues [4]. Despite concerns about *in vivo* degradation and side effects [272], polymeric nanoparticles demonstrate immunocompatibility and hemocompatibility, positioning them as alternatives for intravenous drug administration and making them attractive for potential translation in clinical trials [[273], [274], [275], [276]].

Regulatory agencies such as the FDA and EMA have already approved several nano-based DDSs for ophthalmic use, demonstrating their safety and efficacy. There are currently nine nano-based ophthalmic drugs that have received FDA and/or EMA approval, while several others are undergoing clinical trials in various phases [277]. For instance, Macugen® is the first approved nano-based drug delivery system, utilizing pegaptanib sodium-loaded polyethylene glycol aptamer for the treatment of neovascular age-related macular degeneration. Another notable ophthalmic delivery system is Cequa®, specifically designed for DED. It employs a transparent nano micellar DDS to administer cyclosporine A (CsA), a highly hydrophobic drug [277,278]. The results from these trials have shown promising outcomes, with patients experiencing improved treatment efficacy, prolonged drug action, and enhanced compliance compared to traditional treatment modalities. The ability of polymeric nanoparticles to overcome barriers such as poor ocular penetration, short duration of action, and frequent dosing requirements has been demonstrated in these clinical studies [278]. As new systems with targeted and efficient drug delivery capabilities are being developed, nanomedicine holds the potential to revolutionize the field of ophthalmology and provide new therapeutic options for patients.

The transparency of intraocular structures to a wide range of radiations makes light an attractive external stimulus for exogenously triggered ocular drug delivery applications. Precise spatiotemporal control is crucial for responsive DDSs, especially for the treatment of ocular diseases [25,262]. For instance, the integration of polymeric nanoparticles with photodynamic therapy offers a promising strategy for the treatment of ocular tumours and neovascular diseases [244,279]; photothermal effects have enabled interesting applications of a closely controlled drug delivery for treating ocular pathologies [252,260]. Overall, the development of new ocular drug delivery strategies that enable less invasive and less frequent administrations, effective drug targeting and action, reduced side effects, and improved patient adherence is of the utmost importance [39].

### 6.1 Limitations of PNPs and light irradiation

Despite the promising advancements in PNP for ocular drug delivery, several challenges need to be addressed to ensure their successful translation into clinical practice. The prevalence of ocular diseases is projected to increase significantly in the near future due to factors such as ageing, lifestyle factors, and various health conditions. Thus, the scalability and cost-effectiveness of manufacturing PNP gain high relevance. The production of nanoparticles on a large scale while maintaining batch-to-batch consistency remains an obstacle to meeting the clinical demands and commercial viability of these technologies. Additionally, the cost of production should be considered to ensure the affordability and accessibility of nanoparticle-based ocular therapies [4,39,42]. Nonetheless, the lack of regulatory guidance for ocular drugs complicates the design of nonclinical programs, requiring careful consideration of species, strains, and ocular toxicity parameters [215].

Another significant challenge is represented by regulatory considerations associated with the use of nanotechnology-based DDSs. The regulatory agencies require a comprehensive characterization and safety evaluation of polymeric nanoparticles, including their physicochemical properties, stability, toxicity, and manufacturing processes. The current critical aspects influencing the clinical applicability of final biomedical products are the biodegradability of nanomaterials and the heterogeneity of the final product, which significantly affect biocompatibility, biosafety and therapeutical stability. Biodegradability plays a crucial role in biosafety, cargo release, and retention in targeted ocular cells, while robust manufacturing processes and standardized analytical tools and methodologies are essential for better clinical translation [4,42]. Close collaboration between researchers, clinicians, and regulatory bodies is crucial to streamlining the regulatory approval process and facilitating the clinical translation of these novel therapies [39].

The impact of light irradiation on ocular tissues, spanning various wavelengths, may introduce risks such as oxidative stress, inflammation, and cellular damage, especially with prolonged or intense exposure [280]. UV light, visible light, and blue light exposure have been linked to oxidative damage in ocular tissues, including the cornea, lens, and retina, with the extent of damage influenced by the wavelength and duration of exposure [[281], [282], [283], [284], [285]]. Shorter wavelengths pose a higher risk of inducing photochemical damage to the retina [286,287]. The potential for light exposure to cause DNA damage and impact cellular viability in ocular tissues underscores the need for a comprehensive understanding [288,289]. Although standards for safe exposure limits in ocular tissues exist, further research is essential, particularly regarding the safety of light therapy in individuals with pre-existing ocular abnormalities or increased photosensitivity [290,291]. Thoroughly examining the dosage and duration of irradiation is crucial in designing UV-triggered nanoformulations, as exemplified by Ref. [33].

The interaction of near-infrared (NIR) light with ocular tissues has been extensively studied, particularly in biomedical applications. NIR light's ability to penetrate tissues with reduced light-tissue interaction and increased allowable power density for irradiation offers various applications [292,293]. NIR-induced permeation enhancement has been explored for drug delivery through human sclera, indicating the potential benefits of NIR in ocular drug delivery [294]. NIR light is highlighted as a safe and effective stimulus for triggering drug release from nanoparticles [295].

Concerns about the high temperatures generated following photothermal effects may arise regarding NIR sources. Evaluating the impact of temperature rises in aqueous solutions before in vitro or *in vivo* testing is crucial for studies employing NIR-responsive polymeric nanoformulations. The amount of tolerable heat depends on the application, with some opting for a chemo-photothermal combined treatment of ocular tumours [252,253,259], and others tailoring the photothermal effect to avoid cellular damage [260]. Another example of NIR-triggered release from polymeric nanoparticles with a biocompatible temperature rise is found in Ref. [296].

### 6.2 Future perspectives and concluding remarks

Despite progress, we believe some challenges persist in the clinical translation and commercialization of polymer-based nanoformulations [4]:

o Simplifying nanocarrier construction without compromising bioactivity may be explored to favour clinical translation [4,42].
o Addressing biosafety concerns, including genotoxicity, neurotoxicity and reproductive toxicity, is imperative [4,272]. In vivo biocompatibility issues related to intricately designed nanomaterials have been extensively reviewed by Ref. [272].
o Robust manufacturing processes, standardized analytical tools, and methodologies are crucial for addressing the heterogeneity of nanocarriers and ensuring therapeutic stability [297].
o Further research is essential to bridge the gap between in vitro and *in vivo* evaluations, also through the development of advanced 3D eye tissue models, more predictive of therapeutic responses in humans [298]. Efforts should focus on elucidating the underlying mechanisms of ocular nanomedicine, quantifying nanomaterial reaction kinetics *in vivo*, and addressing biosafety concerns for successful clinical translation [4]. Mathematical modelling may aid in predicting drug release rates and diffusion behaviour from delivery systems and optimizing kinetics by understanding drug transport mechanisms [25].
o Future developments for ocular drug delivery may also include novel approaches that combine the controllable nature of light-responsive systems with the sustained release offered by more complex systems, such as dual delivery platforms consisting of nanoparticle-embedded hydrogels [34]. To the best of our knowledge, there are only a few examples in the literature of dual-release controlled DDSs for ocular applications [243,[299], [300], [301], [302], [303]]. Moreover, the scope of NIR-DDS is anticipated to extend into theranostic nanomedicine, seamlessly integrating therapeutic and diagnostic functionalities.

In conclusion, the recent advancements in the field of PNP for ocular drug delivery have sparked enthusiasm and offered promising solutions to the challenges faced by conventional ocular therapies. The versatility and unique properties of PNP have demonstrated their immense potential in revolutionizing the treatment landscape for a wide range of retinal diseases and disorders. While challenges remain, light-responsive PNPs can significantly improve the treatment landscape for retinal diseases, leading to the goal of a better quality of life for the patients.

## Data availability statement

The authors declare that no data associated with our study has been deposited into a publicly available repository since no data was used for the research described in the article.

## CRediT authorship contribution statement

**Lorenzo Guidi:** Writing – review & editing, Writing – original draft, Resources, Methodology, Investigation, Formal analysis, Conceptualization. **Maria Grazia Cascone:** Writing – review & editing, Methodology, Formal analysis, Conceptualization. **Elisabetta Rosellini:** Writing – review & editing, Supervision, Methodology, Formal analysis, Conceptualization.

## Declaration of competing interest

The authors declare that they have no known competing financial interests or personal relationships that could have appeared to influence the work reported in this paper.
